# The burden of skin and subcutaneous diseases: findings from the global burden of disease study 2019

**DOI:** 10.3389/fpubh.2023.1145513

**Published:** 2023-04-17

**Authors:** Aobuliaximu Yakupu, Rehanguli Aimaier, Bo Yuan, Bin Chen, Jia Cheng, Yaohua Zhao, Yinbo Peng, Jiaoyun Dong, Shuliang Lu

**Affiliations:** ^1^Department of Burn, Ruijin Hospital, Shanghai Jiao Tong University School of Medicine, Shanghai, China; ^2^Wound Healing Center, Ruijin Hospital, Shanghai Jiao Tong University School of Medicine, Shanghai, China; ^3^Department of Plastic and Reconstructive Surgery, Shanghai Ninth People's Hospital, Shanghai Jiao Tong University School of Medicine, Shanghai, China; ^4^Department of Burn and Plastic Surgery, Guangzhou Red Cross Hospital, Jinan University, Guangzhou, China; ^5^Department of Burn and Plastic Surgery, Affiliated Hospital of Jiangnan University, Wuxi, China; ^6^Department of Burn and Plastic Surgery, Jiangyin Hospital Affiliated to Medical College of Southeast University, Jiangyin, China; ^7^Department of Burns and Plastic Surgery, Shanghai Ninth People's Hospital, Shanghai Jiao Tong University School of Medicine, Shanghai, China; ^8^Institute of Traumatic Medicine, Shanghai Jiao Tong University School of Medicine, Shanghai, China

**Keywords:** skin and subcutaneous diseases, Global Burden of Disease, epidemiology, disability-adjusted life years, incidence, gross domestic product, universal health coverage

## Abstract

**Background:**

The small number of existing integrative studies on the global distribution and burden of all types of skin and subcutaneous diseases hinders relevant comparisons.

**Objective:**

This study aimed to determine the latest distribution, epidemiological differences, and factors potentially influencing each skin and subcutaneous disease and the policy implications.

**Methods:**

Data on the skin and subcutaneous diseases were obtained from the Global Burden of Disease Study 2019. The incidence, disability-adjusted life years (DALYs), and deaths due to skin and subcutaneous diseases in 204 countries and regions from 1990 to 2019 were analyzed and stratified by sex, age, geographical location, and sociodemographic index (SDI). The annual age-standardized rate of change in the incidence was obtained to evaluate temporal trends.

**Results:**

Of 4,859,267,654 (95% uncertainty interval [UI], 4,680,693,440–5,060,498,767) new skin and subcutaneous disease cases that were identified, most were fungal (34.0%) and bacterial (23.0%) skin diseases, which accounted for 98,522 (95% UI 75,116–123,949) deaths. The burden of skin and subcutaneous diseases measured in DALYs was 42,883,695.48 (95%UI, 28,626,691.71-63,438,210.22) in 2019, 5.26% of which were years of life lost, and 94.74% of which were years lived with disability. The highest number of new cases and deaths from skin and subcutaneous diseases was in South Asia. Globally, most new cases were in the 0–4-year age group, with skin and subcutaneous disease incidence slightly higher in men than in women.

**Conclusion:**

Fungal infections are major contributors to skin and subcutaneous diseases worldwide. Low–middle SDI states had the highest burden of skin and subcutaneous diseases, and this burden has increased globally. Targeted and effective management strategies based on the distribution characteristics of each country are, thus, required to reduce the burden of skin and subcutaneous diseases.

## 1. Introduction

Skin and subcutaneous diseases, such as acne, alopecia, bacterial skin infections, decubitus ulcers, fungal skin diseases, pruritus, psoriasis, scabies, urticaria, viral skin diseases, and other skin and subcutaneous diseases, are common health problems worldwide and are the leading causes of the global disease burden (1). Skin and subcutaneous diseases can lead to profound long-term alterations even after the disease has resolved, affecting not only the physical health but also the mental health and quality of life of the patient, placing a high burden on patients' families and national healthcare systems globally (2–7).

Knowledge of skin disease epidemiology is essential for policy development, resource allocation, and disease prevention. However, previous studies have mainly focused on specific skin and subcutaneous diseases or countries, which has led to a lack of geographically comparative information on all skin and subcutaneous diseases at different levels (1, 8–14). Therefore, the latest spatial distribution and temporal trends in skin and subcutaneous diseases worldwide must be understood to develop policies to establish more reasonable and effective investigation, prevention, and treatment programs to improve patients' quality of life and reduce avoidable medical expenses.

In the present study, to identify the epidemiological differences between each skin and subcutaneous disease at the national, regional, and global levels, we extracted and analyzed annual data on skin and subcutaneous disease incidence, disability-adjusted life years (DALYs), and deaths by location, sex, and age (15). Our results provide a basis for developing policies and optimizing strategies to manage skin and subcutaneous diseases.

## 2. Materials and methods

We collected the annual case data and age-standardized rates for skin and subcutaneous disease incidence, deaths, and DALYs from 1990 to 2019 from the Institute for Health Metrics and Evaluation using the Global Health Data Exchange online query tool (http://ghdx.healthdata.org/gbd-results-tool) (16). Data on deaths were, however, only available for certain skin and subcutaneous diseases (e.g., bacterial skin diseases and decubitus ulcers).

A detailed introduction of the original data and general analysis methods of the Global Burden of Disease (GBD) Study 2019 have been described previously (15–19). The GBD estimation process was based on identifying multiple relevant data sources for each skin disease and correcting for known biases. The processed data were then modeled using standardized tools to generate estimates for each item of interest based on age, sex, location, and year. The cause-of-death ensemble model was used to analyze the death data of the causes (skin and subcutaneous diseases, bacterial skin diseases, decubitus ulcers, and other skin and subcutaneous diseases), using standard parameters. The years of life lost (YLLs) for each skin disease were calculated by multiplying the number of estimated deaths by the standard life expectancy at the age of death. The years lived with disability (YLDs) for each skin disease were computed by the sequelae, as prevalence was multiplied by disability weights for the health state associated with that sequela. The DALYs of each skin disease were calculated by adding the YLLs and YLDs. Meta-regression (with Bayesian priors, regularization, and trimming models) was used to evaluate the prevalence and incidence of all skin and subcutaneous diseases. Conceptually, the estimation effort of each skin disease was divided into eight major components: (1) compiling data sources through data identification and extraction, (2) data adjustment, (3) estimation of prevalence and incidence by cause and sequelae using meta-regression with Bayesian priors, regularization, and trimming or alternative modeling strategies for selected cause groups, (4) estimation by impairment, (5) severity distributions, (6) incorporation of disability weights, (7) comorbidity adjustment, and (8) estimation of YLDs by sequelae and causes. Age-standardized rates were calculated by adjusting for population size (per 100,000 individuals) and age distribution.

The 95% uncertainty intervals (UIs) for every metric in the GBD Study 2019 were calculated to reflect the certainty of the estimates, which were determined by the 25th and 975th values of the 1,000 values after ordering them from smallest to largest (15, 16, 20). Data extracted from the GBD Study 2019 were collected from 204 countries and territories (data sources including censuses, household surveys, civil registration and vital statistics, disease registries, health service use, air pollution monitors, satellite imaging, disease notifications, and other sources), and were divided into five regions, according to their sociodemographic index (SDI) developed by GBD researchers. This index is a composite indicator constructed from measures of per capita income, average years of education, and total fertility rate. Briefly, the SDI is the geometric mean of the 0 to 1 index of the total fertility rate for those younger than 25 years old, mean education for those 15 years old and older, and lag-distributed income per capita. After calculating the SDI for the GBD Study 2019, the values were multiplied by 100 on a scale of 0–100. Geographically, the 204 countries and territories are classified into 21 regions based on their locations. The annual age-standardized incidence rate of change was used to estimate disease trends.

To explore the potential impact factors of changing trends, we also calculated the association between universal health coverage (UHC) and gross domestic product (GDP) and the annual age-standardized incidence rate of changes in skin and subcutaneous diseases. The UHC was estimated using the UHC effective coverage index, which provides an understanding of whether health services are aligned with the countries' health profiles and are of adequate quality to produce health gains for populations (24). We then consider the policy implications of the analyzed data.

### 2.1. Data analysis

The correlations of the annual age-standardized incidence rate of change with UHC in 2019, GDP in 2019, and SDI value, and the correlations between skin diseases were determined using Pearson correlation analysis (21). All analyses were performed using R software, version 4.2.1, with two-sided *p* < 0.05 indicating a statistically significant difference.

## 3. Results

### 3.1. General status of global skin and subcutaneous diseases burden

**Figure 1:**
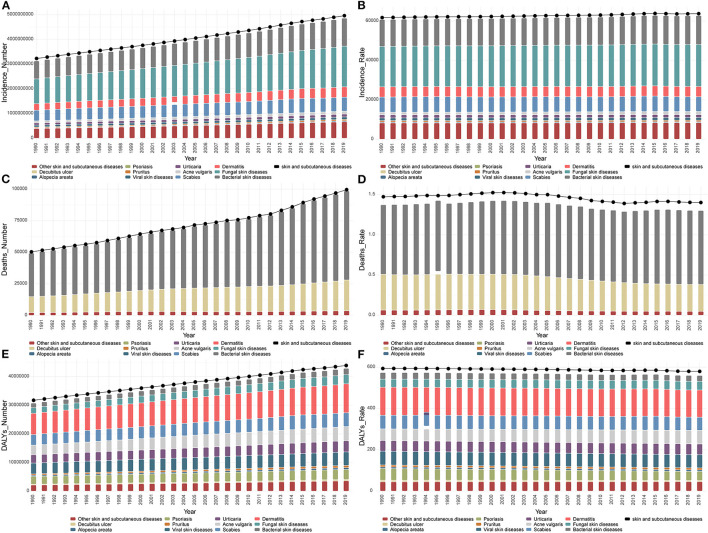
General status of global skin and subcutaneous diseases burden. **(A)** New cases of each skin and subcutaneous disease globally from 1990 to 2019. **(B)** ASIR of each skin and subcutaneous disease globally from 1990 to 2019. **(C)** Deaths from each skin and subcutaneous disease globally from 1990 to 2019. **(D)** ASDR from each skin and subcutaneous disease globally from 1990 to 2019. **(E)** DALYs from each skin and subcutaneous disease globally from 1990 to 2019. **(F)** ASDAR from each skin and subcutaneous disease globally from 1990 to 2019. ASIR, age-standardized incidence rate; DALYs, disability-adjusted life years; ASDAR, age-standardized DALYs rate; ASDR, age-standardized death rate.

Globally, 4,859,267,654 new skin and subcutaneous disease cases (95% UI 4,680,693,440–5,060,498,767 cases) were identified in 2019, most of which were fungal and bacterial skin diseases. These accounted for 34% and 23%, respectively [fungal cases: 1,646,596,956 (95% UI 1,493,240,311–1,811,901,501); bacterial: 1,134,138,000 (95% UI 1,105,834,933–1,169,419,691) cases] (Figure 1A and Supplementary Table S1). In total, 98.522 (95% UI, 75.116.11–123.948.56) people died from skin and subcutaneous diseases globally in 2019. This was twice the number in 1990, and most died due to bacterial skin diseases (72%) (Figure 1C and Supplementary Table S2). The highest number of new cases and deaths due to skin and subcutaneous diseases were recorded in South Asia (new cases, 25%; deaths, 25%), and, especially in India (new cases,19%; deaths,25%) (Figures 1A, C and Supplementary Tables S1, S2). The burden of skin and subcutaneous diseases measured in DALYs in 2019 was 42,883,695.48 (95%UI, 28626,691.71–63,438,210.22), of which 5.26% and 94.74% could be attributed to YLLs and YLDs, respectively. In 2019, most DALYs were caused by bacterial skin diseases (23%) and were concentrated in South Asia (21%), particularly China (19%) (Figure 1E and Supplementary Table S3). From 1990 to 2019, the new and deaths of all types of skin and subcutaneous diseases and their burden measured in DALYs increased substantially. The new cases and DALYs of decubitus ulcers and the number of deaths due to bacterial skin diseases increased from 1990 to 2019 by 106%, 80%, and 104% (Figures 1A, C, E).

For the age-standardized rates, the skin and subcutaneous diseases associated with the highest recorded age-standardized incidence rate (ASIR), age-standardized DALYs' rate (ASDAR), and age-standardized death rate (ASDR) in 2019 were fungal skin diseases (21,276.58 [95% UI, 19,297.52–23,398.73]), dermatitis (131.67 [95% UI, 77.59–206.87]), and bacterial skin diseases (0.92 [95% UI, 0.67–1.14]) (Figures 1C, D, F). Overall, the ASDAR and ASDR tended to decrease with slight fluctuations, whereas the ASIR continued to increase from 1990 to 2019 (Figures 1C, D, F). However, from the perspective of different skin and subcutaneous diseases, the ASIR of scabies, alopecia areata, psoriasis, decubitus ulcers, and viral skin diseases decreased, and the ASDAR of acne vulgaris, fungal skin diseases, pruritus, urticaria, other skin, and subcutaneous diseases, and the ASDR of bacterial skin diseases increased. The skin and subcutaneous diseases that showed a significant increase in the ASIR, ASDAR, and ASDR were acne vulgaris (18%), acne vulgaris (16%), and bacterial diseases (6%).

### 3.2. Skin and subcutaneous diseases burden among the different sexes and ages

**Figure 2:**
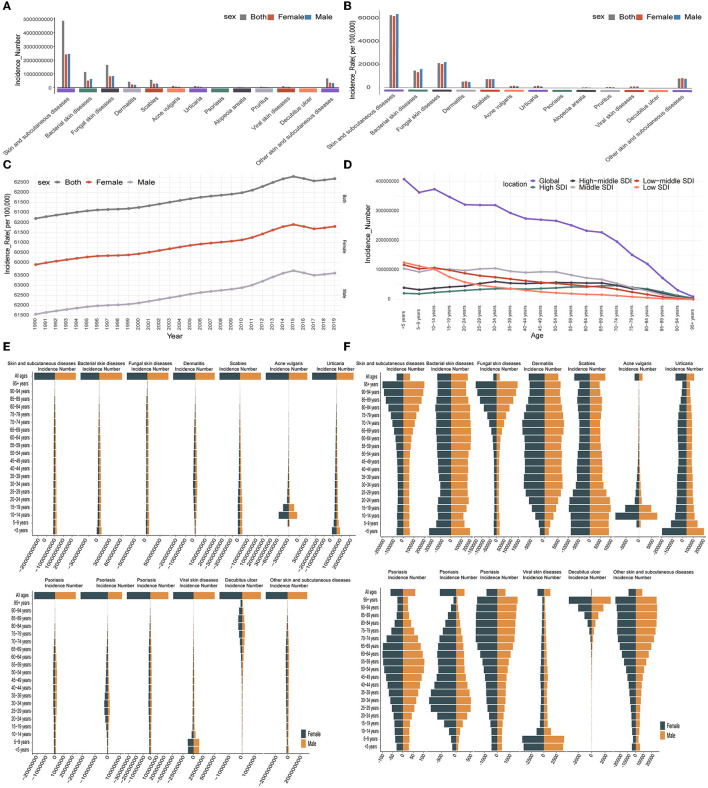
Skin and subcutaneous diseases burden among different sexes and ages. **(A)** Each skin and subcutaneous disease's new cases distributions by sex in 2019. **(B)** Each skin and subcutaneous disease ASIR distribution by sex in 2019. **(C)** Global changing trend in each skin and subcutaneous disease ASIR by sex from 1990 to 2019. **(D)** Distribution of all skin and subcutaneous diseases new cases globally and SDI levels among different age categories in 2019. **(E)** Distribution of each skin and subcutaneous disease new cases among different age categories in 2019. **(F)** Distribution of each skin and subcutaneous disease incidence rate among different age categories in 2019. ASIR, age-standardized incidence rate; SDI, sociodemographic index.

Globally, in 2019, the new cases of skin and subcutaneous diseases in men were slightly higher than in women (men: 2,446,443,162.05 [95%UI, 2,349,576,424.39–2,548,351,122.13]; women: 2,412,824,491.40 [95%UI, 2,323,159,191.20–2,509,074,646.14]) (Figure 2A). Fungal skin diseases were the most common skin diseases in both sexes but tended to occur more frequently in men; their ASIR was the highest for both sexes, especially in men (22,075.82, 95%UI [19,998.15–24,357.26]) (Figure 2B). Based on 2019 data, the disease with the greatest difference in incidence between men and women is alopecia areata, which is more common in women. Dermatitis was also more common in women, and their ASIR was higher (Figures 2A, B). The skin and subcutaneous diseases that were more common in men were bacterial skin and subcutaneous diseases and scabies, whereas, in women, scabies, acne vulgaris, urticaria, alopecia areata, pruritus, and decubitus ulcers were more common. Scabies was notably prevalent in both sexes (Figure 2A). Viral skin diseases and psoriasis were almost evenly distributed among men and women (Figure 2A). The DALYs among women were slightly higher than those among men, and the ASDAR among women was also slightly higher than that among men in 2019 (Supplementary Figures S1A, B). Dermatitis contributed to the highest DALYs among the skin and subcutaneous diseases in both sexes and was associated with the highest recorded ASDAR in women (151.79 [95%UI, 89.09–239.06]) (Supplementary Figures S1A, B). The number of deaths from skin and subcutaneous diseases was higher in women than in men in 2019, whereas the ASDR was higher in men (Supplementary Figures S1C, D). Bacterial skin diseases accounted for the highest number of deaths among skin and subcutaneous diseases in both sexes, particularly in women (Supplementary Figures S1C, D).

From 1990 to 2019, the proportion of men and women with each type of skin and subcutaneous disease did not change significantly (Supplementary Figure S2A). During this period, the number of new cases, DALYs, and deaths increased substantially each year in both sexes (Supplementary Figures S2B, C, E). Compared to 1990, the types of skin and subcutaneous diseases that increased the most in new cases (108%), deaths (96%), and DALYs (80%) in men were decubitus ulcers, while decubitus ulcers also increased the most in women with increased new cases (103%) and DALYs (79%) (Supplementary Table S4). However, the types of skin and subcutaneous diseases that increased the most concerning deaths in women were bacterial skin diseases, which increased by 119% (Supplementary Table S4). With age-standardized rates, the ASDAR and ASDR tended to decrease with slight fluctuations, whereas the ASIR increased in both sexes (Figure 2C and Supplementary Figures S2D, F). However, the ASIR and ASDAR increased or decreased for different skin and subcutaneous diseases. In both sexes, the diseases that increased or decreased in the ASIR or ASDAR were the same as those at the global level as described previously. For ASDR, only the ASDR of bacterial skin diseases increased in women, whereas the other diseases decreased in both sexes (Supplementary Table S4). From the perspective of sex, the skin and subcutaneous diseases that showed the greatest increases in ASIR, ASDAR, and ASDR were acne vulgaris (men: 21%; women: 21%), acne vulgaris (men: 15%; women: 13%), and bacterial skin diseases (women: 13%), respectively (Supplementary Table S4).

Skin and subcutaneous diseases were more common in young patients, and most new cases were in the 0–4-year age group in 2019 (Figure 2D). DALYs were also high in the younger population, and the highest DALYs were observed in the 15–19-year age group; however, deaths were concentrated in those aged 80+ years for both sexes (Supplementary Figures S3B, D). ASIR, ASDAR, and ASDR were all higher in older individuals in 2019 (Supplementary Figures S3A, C, E). In addition, scabies, acne vulgaris, urticaria, and viral skin diseases were more common in the younger generation and contributed to the highest DALYs, while decubitus ulcers were more common in older individuals, contributing to the highest DALYs and deaths (Figure 2E and Supplementary Figures S4A, B). Fungal skin diseases, pruritus, and decubitus ulcers demonstrated significantly higher ASIR and ASDAR in older patients, whereas scabies, urticaria, and viral skin diseases are associated with high ASIR and ASDAR values in young individuals. In addition, bacterial skin diseases were associated with significantly higher ASDAR and ASDR values in older individuals (Figure 2F and Supplementary Figures S4C, D). From 1990 to 2019, there was little change in the age distribution of all the skin and subcutaneous diseases (Supplementary Figure S3F).

### 3.3. Skin and subcutaneous diseases burden among different social-economic status

**Figure 3:**
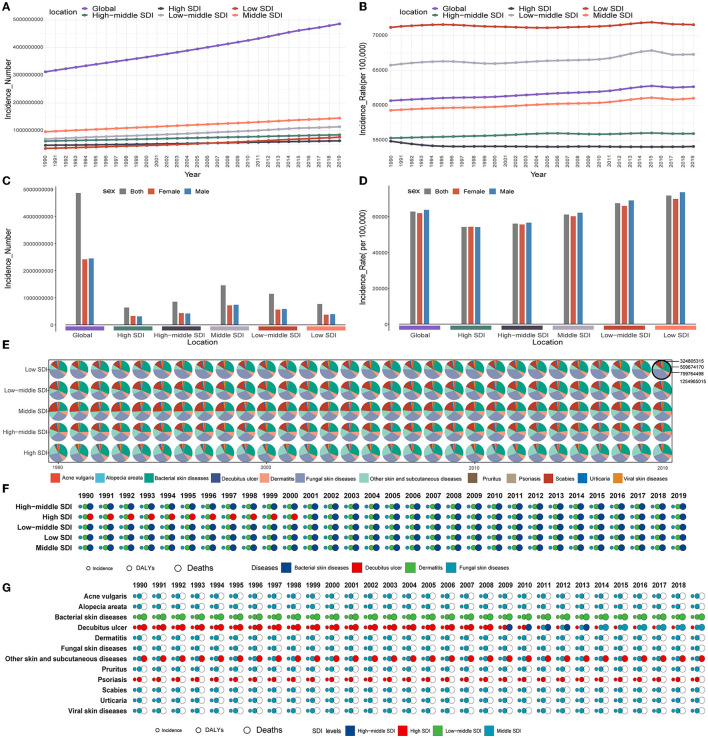
Skin and subcutaneous diseases burden among different social–economic states. **(A)** Changing trend in each skin and subcutaneous disease new cases by global and SDI levels from 1990 to 2019. **(B)** Changing trend in each skin and subcutaneous disease ASIR by global and SDI levels from 1990 to 2019. **(C)** Skin and subcutaneous diseases new cases distribution by sex at different SDI levels in 2019. **(D)** Skin and subcutaneous diseases ASIR distribution by sex at different SDI levels in 2019. **(E)** The proportion of new cases of each skin and subcutaneous disease at different SDI levels from 1990 to 2019. **(F)** Type of skin and subcutaneous diseases that contribute the highest new cases, DALYs, and deaths at different SDI levels from 1990 to 2019. **(G)** SDI levels recorded the highest new cases, DALYs, and deaths of each skin and subcutaneous disease from 1990 to 2019. ASIR, age-standardized incidence rate; DALYs, disability-adjusted life years; SDI, sociodemographic index.

From the perspective of the different SDI levels, countries with middle SDI levels had the highest number of new cases of skin and subcutaneous diseases (1,456,208,410 [95%UI, 1,399,357,076–1,514,043,198]), accounting for 30% of the global new cases in 2019, and had the highest number of new cases from 1990 to 2019 (Figure 3A). The number of new cases increased for all the SDI levels from 1990 to 2019; it significantly increased for the low SDI level (in 2019: 766,636,889.27 [95%UI, 721,990,381.15–814,942,421.22]), and the increase was 113% (Figure 3A). The highest ASIR was recorded for the low SDI level in 2019 (71,593.23, 95%UI [68,370.55–75,077.99]), but the ASIR increased for all SDI levels except the high SDI level from 1990 to 2019; the highest increase for those years was found at the middle SDI level (61,038.54 [95%UI, 58,612.56–63,536.31]), an increase of 3% (Figure 3B). Skin and subcutaneous diseases were more common in the young age groups for the lower SDI levels. However, they were more common in older patients at higher SDI levels, and the ASIR was higher in older individuals at all SDI levels in 2019 (Figure 2D and Supplementary Figure S3A). The number of new cases was higher in women than in men with higher SDI levels, whereas it was higher in men with lower SDI levels in 2019 (Figure 3C). Except for the high SDI level, the ASIR was higher in men at all SDI levels in 2019 (Figure 3D).

In 2019, the highest DALYs were recorded for the middle SDI level [13,062,128.19 (95%UI, 8,598,805.41–19,549,276.27]). The DALYs increased for all SDI levels from 1990 to 2019, and the middle SDI level had the highest DALYs compared to the other SDI levels in this same period, whereas the DALYs significantly increased (by 110%) at the low SDI level (In 2019: 5,956,125.74 [95%UI, 3,925,718.84–8,999,847.35]) (Supplementary Figure S5A). From 1990 to 2019, the highest ASDAR was recorded at the high SDI level, and it significantly decreased at all SDI levels, except for the high-middle SDI level (Supplementary Figure S5B). The DALYs were also high in the younger population, whereas the ASDAR was higher in the older population at all SDI levels (Supplementary Figures S3B, C). Concerning the sex ratios of the DALYs and ASDARs among SDI levels, these were higher in women at all SDI levels in 2019 (Supplementary Figures S5C, D).

In 2019, the highest number of deaths was recorded at the middle SDI level (25,814.65, 95%UI [21,781.51–30,732.72]). Deaths increased at all SDI levels from 1990 to 2019, and the middle SDI level had the highest number of deaths at the same time, whereas the number of deaths significantly increased at the high-middle SDI level (In 2019: 20,014.62 [95%UI, 13,431.51–26,288.53]), with an increase of 138% (Supplementary Figure S5E). From 1990 to 2019, the highest ASDR was recorded at the low–middle SDI level. The ASDR decreased at lower SDI levels and increased at higher SDI levels (Supplementary Figure S5F). The deaths were concentrated in those aged 80+ years, and the ASDR was also recorded to be higher in the older population at all SDI levels (Supplementary Figures S3D, F). In terms of sex, the number of women who died was higher than men at all SDI levels in 2019. However, at higher SDI levels, the ASDR values were higher in men than in women in 2019 (Supplementary Figures S5G, H).

At all SDI levels, the skin and subcutaneous diseases that contributed to the highest number of new cases, DALYs, and deaths were fungal skin diseases, dermatitis, and bacterial skin diseases, respectively (Figures 3E, F and Supplementary Figures S6A, B). Except for bacterial skin diseases and psoriasis, and the new cases of the decubitus ulcer, the SDI level associated with the highest number of new cases, DALYs, and deaths of different skin and subcutaneous diseases were all at the middle SDI level (Figure 3G). At all SDI levels, the skin and subcutaneous diseases that contributed the highest ASIR, ASDAR, and ASDR were fungal skin diseases, dermatitis, and bacterial skin diseases, respectively (Supplementary Figures S6C–F). The SDI values associated with the highest recorded ASIR, ASDAR, and ASDR values for each skin and subcutaneous disease are shown in Supplementary Figure S6G.

### 3.4. Skin and subcutaneous diseases burden in different regions

The absolute number of new cases, DALYs, and deaths due to skin and subcutaneous diseases increased in almost all GBD regions. South Asia had the highest number of new cases, DALYs, and deaths in 2019 (incidence: 1,208,952,415.44 [95% UI 1,162,257,056.17–1,259,536,849.02]; DALYs: 8,965,385.45 [95%UI, 5,949,896.33–13,202,307.74]; deaths: 8,965,385.45 [95%UI, 5,949,896.33–13,202,307.74]), which increased by 55%, 45%, and 71%, respectively, from 1990 and accounted for 21%, 20%, and 25% of the total global new cases, DALYs, and deaths, respectively. However, Oceania had the fewest new cases of DALYs in 2019 (incidence: 7,826,121.01 [95% UI, 7,387,971.02–8,259,634.86]; DALYs: 88,973.81 [95%UI, 58,072.08–129,933.28]) and the lowest number of deaths recorded in Central Asia (166.76 [95% UI, 114.60–249.40]). From 1990 to 2019, Western Sub-Saharan Africa exhibited the highest percentage increase in new cases and DALYs, increasing by 137% and 140%, respectively (in 2019, incidence: 336,459,662.65 [95% UI, 313,416,523.10–361,249,639.71]; DALYs: 2,329,711.49 [95% UI, 1,543,480.32–3,570,126.42]). The most significant increase in deaths detected in Tropical Latin America was 363% (1,639.44 [95% UI, 1,134.41–2,772.35] to 7,592.79 [95% UI, 3,452.25–9,329.05]).

**Figure 4:**
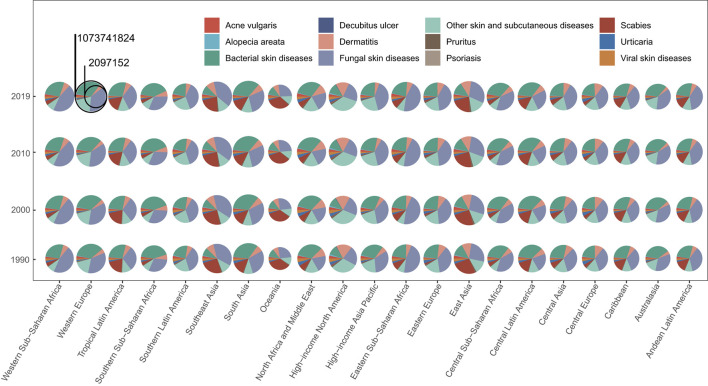
Skin and subcutaneous diseases burden in different regions. The proportion of new cases of each skin and subcutaneous disease at different GBD regions (21) in 1990, 2000, 2010, and 2019. GBD, Global Burden of Disease.

From 1990 to 2019, the skin and subcutaneous diseases that contributed the most to the incidence in each region were mostly fungal skin diseases, followed by bacterial skin diseases, in which pyoderma was the most prevalent (Figure 4). During the same period, the greatest contributors to DALYs and deaths in each region were dermatitis and bacterial skin diseases, respectively (Supplementary Figures S7A, B, S8A). The highest number of acne vulgaris, alopecia areata, dermatitis pruritus, scabies, viral skin diseases, and other skin and subcutaneous diseases was recorded in East Asia; the highest number of bacterial, fungal skin diseases, and urticaria was recorded in South Asia; the highest number of decubitus ulcers was recorded in high-income North America; and the highest number of decubitus ulcers was recorded in high-income North America. Except for acne vulgaris, decubitus ulcer, and viral skin diseases, the regions associated with the highest recorded DALYs and deaths associated with each skin and subcutaneous disease were the same as the regions that recorded the highest number of new cases; the regions associated with the highest recorded DALYs of acne vulgaris, decubitus ulcer, and viral skin diseases were East Asia, Southeast Asia, and East Asia, while the highest number of decubitus ulcers was found in Southeast Asia (Supplementary Figure S8B). The most geographically differentiated skin and subcutaneous disease is scabies, which is mainly distributed in East, South, and Southeast Asia and in Andean, Central, and Latin America, and is also the most common type of skin and subcutaneous disease in Oceania (Supplementary Figure S9).

From 1990 to 2019, the ASIR, ASDAR, and ASDR increased in most regions. Southern Sub-Saharan Africa had the highest ASIR in 2019 (80,648.03 [95% UI, 77,453.40–84,092.88]), whereas high-income North America had the lowest ASIR (37,643.70 [95% UI, 36,577.88–38,763.59]). The greatest percentage reduction detected was in Central Europe (from 55,172.28 [95% UI, 52,989.80–57,557.31] to 54,784.19 [95% UI, 52,593.09–57,195.97]), whereas the most significant increase was detected in Central Latin America (from 63,994.58 [95% UI, 66,214.65–61,814.18] to 65,539.00 [95% UI, 63,387.70–67,847.44]). The highest ASDAR was observed in 2019 in Western Europe (739.59 [95%UI, 505.92–1,047.66]) and the lowest was in North Africa and the Middle East (419.87 [95%UI, 282.04–618.05). The most significant decrease in ASDAR was detected in high-income North America (from 681.57 [95% UI, 472.83–952.48] to 655.73 [95% UI, 455.25–918.96]), whereas the most significant increase was detected in Tropical Latin America (from 623.14 [95% UI, 420.53–918.43] to 650.68 [95% UI, 443.80–939.85]). Southern Latin America had the highest ASDR in 2019 and the most significantly increased ASDR among the 21 GBD regions (from 1.86 [95% UI, 1.27–4.19] to 3.61 [95% UI, 2.14 −5.31]). Central Asia had the lowest ASDR in 2019 (0.25 [95% UI, 0.17–0.36]) and the most significant decrease in ASDR was detected in East Asia (In 2019: 0.46 [95% UI, 0.39–0.57]). In most GBD regions, fungal and bacterial skin diseases had the highest ASIR, dermatitis had the highest ASDAR, and bacterial skin and subcutaneous diseases had the highest ASDR (Supplementary Figures S10A–C). Detailed information about the contribution of each disease concerning age-standardized rates in every region and the highest age-standardized rates for each disease was recorded and the information as to which region can be seen in Supplementary Figures S8C, D.

The new cases and the DALYs' distribution of skin and subcutaneous diseases by sex and age in the GBD regions are generally consistent with the global situation (Supplementary Figures S11A, B). However, in Central and Eastern Europe, high-income North America, and Southern Latin America, the proportion of female patients increased significantly, whereas the proportion of male patients increased significantly in North Africa, the Middle East, Oceania, and South Asia (Supplementary Figures S11A, B). The skin and subcutaneous diseases with large regional differences regarding the new cases in men and women were acne vulgaris and decubitus ulcers (Supplementary Figures S11A, B). Compared with the global age distribution of skin and subcutaneous diseases, patients with all skin and subcutaneous diseases tend to be younger in Central, Eastern, and Western Sub–Saharan Africa, whereas patients tend to be older in Australasia, Central Europe, High–income Asia Pacific, High–income North America, Tropical Latin America, and Western Europe (Supplementary Figures S11C, D). The diseases for which the age distribution varied greatly among the regions were bacterial skin diseases, fungal skin diseases, scabies, and urticaria (Supplementary Figure S11C). Concerning the distribution of skin and subcutaneous diseases by sex and age in the GBD regions of Asia, Central, Eastern, and Southern Sub-Saharan Africa, Southern Latin America, and Western Europe, the proportion of female patients increased significantly, while the proportion of male patients increased significantly in Oceania (Supplementary Figure S11E). Patients with all skin and subcutaneous diseases in Central, Eastern, and Western Sub-Saharan Africa tended to be younger (Supplementary Figure S11F). A detailed description of the diseases from the perspective of countries and territories can be found in the Supplementary material, which enable policymakers to address the particular skin and subcutaneous diseases affecting their populations.

### 3.5. Trend of skin and subcutaneous diseases and their relevance with social-economic states

**Figure 5:**
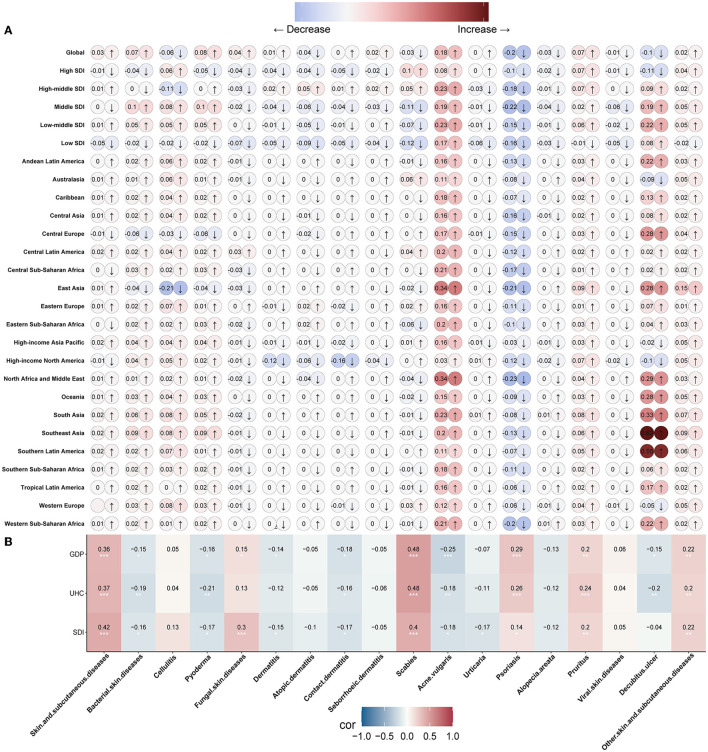
The trend of skin and subcutaneous diseases and the relevance with social-economic states. **(A)** Trends of each skin and subcutaneous disease at global, different SDI levels, and different GBD regions (21). **(B)** Correlation analysis of each skin and subcutaneous disease annual age-standardized incidence rate of changes with SDI, UHC, and GDP among 204 countries and territories in 2019. GBD, Global Burden of Disease; SDI, sociodemographic index; UHC, universal health coverage; GDP, gross domestic product.

We analyzed the temporal trends of different skin and subcutaneous diseases at the regional, SDI, and global levels using the annual age-standardized rate of change in incidence. The results showed that skin and subcutaneous diseases are anticipated to increase globally, especially in low–middle SDI states (Figure 5A). Most skin and subcutaneous diseases exhibited different trends at different levels, whereas acne vulgaris and psoriasis tended to increase and decrease, respectively, at all levels (Figure 5A). Temporal trends of different skin and subcutaneous diseases at the national level are shown in the Supplementary Figure S12.

The socioeconomic status of each nation can be evaluated by the SDI and/or GDP, and UHC is one of the indices used to estimate countries' healthcare system performance (22, 23). To better understand the distribution of skin and subcutaneous diseases based on the SDI, GDP, and healthcare system performance of countries, we performed a correlation analysis between the SDI, GDP, and UHC values of 204 countries/territories in 2019 and the annual age-standardized rate of change in incidence. The SDI, GDP, and UHC values were negatively correlated with most skin and subcutaneous diseases' annual age-standardized rate of change in incidence, indicating that the incidence of most diseases declined with increasing SDI, GDP, and UHC values, suggesting that the SDI, GDP, and UHC indices have a vital impact on the temporal trends of skin and subcutaneous diseases (Figure 5B). However, the annual age-standardized rate of change in the incidence of some skin and subcutaneous diseases, such as fungal skin diseases, psoriasis, pruritus, and scabies, was positively correlated with the SDI, GDP, and UHC. This indicates that some skin and subcutaneous diseases will become increasingly prevalent in high socioeconomic states. Furthermore, we analyzed the relationship between skin and subcutaneous diseases and skin cancer, using the annual age-standardized rate of change in incidence. The results showed that psoriasis negatively correlated with acne vulgaris (r=-0.77), urticaria positively correlated with alopecia areata (r= 0.62), and viral skin diseases positively correlated with seborrheic dermatitis (r= 0.55) (Supplementary Figure S13).

## 4. Discussion

In this study, we systematically assessed the burden of each skin and subcutaneous disease in terms of national, regional, global, sex, age, and socioeconomic status, including dermatitis (atopic, contact, and seborrheic), bacterial skin diseases (cellulitis and pyoderma), scabies, fungal skin diseases, viral skin diseases, acne vulgaris, alopecia areata, pruritus, urticaria, decubitus ulcers, and other skin and subcutaneous diseases. This study revealed the disparity in skin and subcutaneous disease burdens among different regions and countries.

The nations that recorded the highest number of skin and subcutaneous diseases were mostly India and China, mainly because of their large populations. The skin and subcutaneous diseases contributing the highest burden in most regions are fungal skin diseases, followed by bacterial skin diseases. Fungal skin diseases (such as dermatophytosis, subcutaneous opportunistic mycoses, talaromycosis, emergomycosis, and cutaneous phaeohyphomycosis), can occur not only as primary diseases but also because of the dissemination of systematic infections, and present with a diverse spectrum of infection extent, severity, and features (24). They have an ever-growing incidence in or outside their endemic areas, because of the increasing number of immunocompromised patients and worldwide traveling (25, 26). Bacterial skin diseases, such as cellulitis and pyoderma, have also been increasing (27). Moreover, fungal infections are a risk factor for cellulitis (28). Bacterial skin diseases were the main contributors to deaths, indicating that fungal infections can increase the number of deaths. We also found that the ASIR of fungal skin diseases were higher in older individuals, which may be due to age-related anatomical, physiological, and environmental factors that increase their susceptibility to skin infections (29). Therefore, we need to pay more attention should be paid to older adults with fungal or bacterial infections.

Alopecia areata is a common inflammatory disease; however, its cause remains incompletely understood (30, 31). Although previous studies have reported that alopecia areata affects both sexes equally or men more commonly, we found it to be more common in women (32, 33). This may be because an unidentified factor makes women susceptible to alopecia areata, or the prevalence of the subtype of alopecia areata, such as acute diffuse and total alopecia Marie-Antoinette syndrome, mostly in women's scalps (32, 34), is increasing. Unfortunately, the GBD Study 2019 did not provide data for each subtype of alopecia areata. As previously reported, scabies, acne vulgaris, and urticaria are more common in the young (35–37). Although we found that the combined incidence of viral skin diseases was higher in the young, several subtypes of viral skin diseases were observed mostly in older adults (38, 39). Decubitus ulcers are more common in older individuals and contribute to the highest number of deaths, possibly because they are primarily associated with chronic health conditions, and their global prevalence is increasing globally (40, 41).

The number of each type of skin and subcutaneous disease Increased from 1990 to 2019 worldwide; however, the ASIR of different types of skin and subcutaneous diseases exhibited different trends. The ASIR of decubitus ulcers, alopecia areata, psoriasis, viral skin diseases, and scabies decreased, while those of others increased globally, which highlights the remarkable achievements in preventing these diseases. Skin and subcutaneous diseases are common in patients with lower SDI scores, and most of their incidences are negatively correlated with SDI, GDP, and UHC. However, psoriasis, pruritus, and scabies are becoming increasingly prominent in high socioeconomic states. This may be due to the interplay between many factors, such as increased risk factors, differences in lifestyle and cultural perceptions, and overcrowding (1, 42, 43). The annual age-standardized incidence rate of change of most skin and subcutaneous diseases is negatively correlated with UHC, indicating that UHC has a vital impact on disease prevalence.

Moreover, public health policy is an important determining factor for access to healthcare, as it can influence aspects of health service delivery such as the availability of resources, organization, and financing (44). Healthcare policies for skin diseases vary by country and depend on factors such as the prevalence, severity, cost, and availability of treatments for different skin conditions. For example, in India, most dermatological or skin problems are covered by health insurance plans that can help patients afford treatment. However, some skin conditions, such as vitiligo, may not be covered by insurance owing to social stigma or a lack of awareness (11). In South Korea, the National Health Insurance Service covers most dermatological services and drugs; however, some cosmetic procedures or treatments that are not considered medically necessary may require out-of-pocket payments. In the United Kingdom, the National Health Service provides free dermatology services and prescriptions for skin diseases to all residents; however, there may be long waiting times or limited access to specialized treatments or referrals. In Brazil, the Unified Health System offers universal and free access to dermatology services and medications for skin diseases; however, there are challenges such as insufficient funding, human resources, and infrastructure. Generally speaking, these healthcare policies have benefited numerous people, for example, in Sub-Saharan Africa, where HIV-related skin problems are prevalent, a series of disease prevention policies and, in particular, in many countries the availability of free anti-retroviral drugs have significantly reduced the number of deaths and improved the quality of life (45).

However, skin diseases do not receive due importance in national health planning and policies in several countries, and there may be gaps or challenges in accessing quality and affordable care for skin conditions (46). No single best policy suits all countries because each country has its own needs, resources, and priorities. However, according to the World Health Organization (WHO), some common elements that may improve healthcare policies for skin diseases include increasing awareness and education about skin diseases among the public and healthcare providers and integrating approaches for the prevention, diagnosis, treatment, and management of skin diseases across different levels of the healthcare system. The WHO suggests that countries strengthen surveillance and data collection concerning the burden and impact of skin diseases; promote research and innovation for new diagnostics, therapies, and interventions for skin diseases; and enhance collaboration and coordination among stakeholders such as governments, health organizations, civil society groups, and patients. Furthermore, the integration of management strategies has the potential to significantly and cost-effectively bolster health systems in resource-poor areas, leading to improvements in the skin and overall health of many of the world's most disadvantaged individuals (47). Skin diseases affect people's quality of life, self-esteem, social relationships, and mental health. Providing support groups or online forums for people with skin diseases can help reduce feelings of isolation, stigma, or shame associated, for example, with acne vulgaris by sharing experiences, challenges, and coping strategies with others who understand what they are experiencing (48). Additionally, public health policies should timeously adjust to changes in disease risk factors and patterns. For example, with population growth and aging, the number of chronic skin wounds is increasing significantly, and China is accelerating the development of wound repair departments and relevant policies for managing chronic skin wounds (49). With the increase in global communication, it is necessary to develop a global control program for skin diseases that will effectively help measure and reduce the burden of skin diseases worldwide (50–52). Other actions such as increasing awareness and education about skin diseases and their prevention among the public and healthcare workers are suitable for countries with limited funding, especially low- and middle-income countries (14, 53). Local governments struggle to provide adequate medicines and laboratory facilities to improve the diagnosis and treatment of skin diseases. They can set up publicly available webpages to provide information on common skin diseases, raise awareness, and encourage the early detection of skin diseases through media campaigns and community events. Our study indicates that the prevalence of different skin diseases varies from country to country. Strengthening surveillance and data collection on skin disease prevalence on a global scale will help establish more reasonable and effective investigation, prevention, and treatment programs. Thus, it is necessary to establish standardized disease surveillance and data collection procedures and protocols. Additionally, it is necessary to develop a unified system for disease diagnosis and registration. In addition, research resources for different skin diseases should be directed more toward countries where the different types of skin diseases are prevalent, which will enable the effective use of resources and can help obtain more convincing research results in a shorter time because of the large number of patients. Our study also highlights the importance of an integrated public health policy in different countries to promote public health more effectively. Globally, guidance for skin disease control is indispensable.

From our correlation analysis, psoriasis was negatively correlated with acne vulgaris, and the ASIR decreased with increasing acne ASIR. Psoriasis and acne vulgaris are both immune-mediated inflammatory diseases; however, psoriasis is mainly mediated by IL-17 and IL-23, and acne vulgaris by IL-1 (36, 54), which may result in unique inflammatory circuits that promote different pathological processes. In addition, urticaria is positively correlated with alopecia areata, and viral skin diseases are positively correlated with seborrheic dermatitis, all of which are inflammatory diseases. Although the pathogenic relevance of different skin diseases may be determined by further in-depth genetic analyses and studies, the results provide new insights into skin disease investigation and emphasize the importance of integrative investigation, prevention, and treatment of different skin diseases.

### 4.1. Limitations

This study has limitations that are generally associated with GBD studies. First, the accuracy of GBD estimation depends largely on the quality and quantity of data used, as it is associated with underreporting and underdiagnosis during skin and subcutaneous disease registration. Second, the data could not be explored further to extract information related to the subtype of each skin and subcutaneous disease, severity, and treatment because such information was not provided by the Global Health Data Exchange. Therefore, we could not obtain a detailed etiological understanding of the global changes in the skin and subcutaneous disease patterns. Finally, owing to the observational nature of this analysis, some unmeasured confounding factors may not have been discussed, and causal statements could not be made about the trends observed.

### 4.2. Conclusion

**Figure 6:**
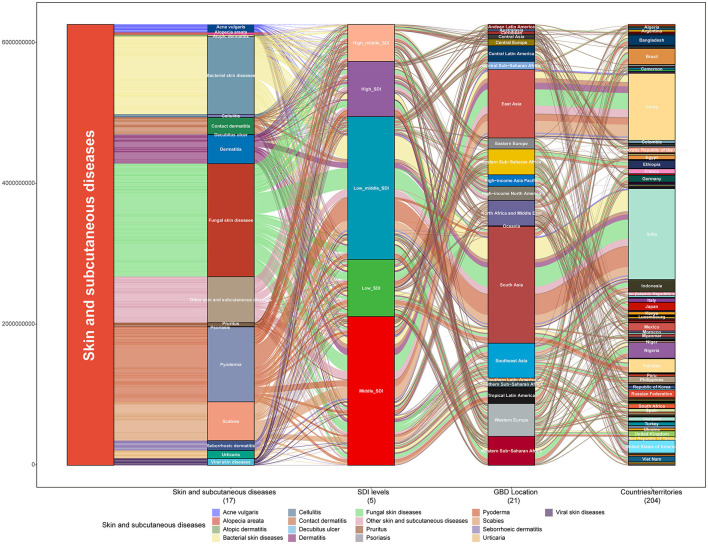
Skin and subcutaneous disease burden in 2019. Distribution of new cases of each skin and subcutaneous disease at the global level, showing different SDI levels, different GBD regions (21), and 204 countries/territories in 2019. GBD, Global Burden of Disease; SDI, sociodemographic index.

Fungal skin diseases are the major contributors to skin and subcutaneous diseases worldwide. The low–middle SDI states had the highest burden of skin and subcutaneous diseases, especially India (Figure 6). A significant negative correlation was observed between psoriasis and acne vulgaris. The burden of skin and subcutaneous diseases is increasing globally. Thus, targeted and effective management strategies are required globally based on the latest distribution characteristics of each country or territory and focused efforts to reduce the burden of skin and subcutaneous diseases are required.

## Data availability statement

Publicly available datasets were analyzed in this study. This data can be found at: The datasets analyzed during the current study are available in the GBD 2019 repository, which is freely available at http://ghdx.healthdata.org/gbd-results-tool.

## Ethics statement

Requirement for ethical approval was waived by the Ethics Committee of Ruijin Hospital, Shanghai JiaoTong University School of Medicine because the results of GBD 2019 study are available in a publicly available database, and all data were anonymous. All methods were performed according to the relevant guidelines and regulations.

## Author contributions

AY and RA analyzed and visualized the data. AY, RA, JD, and SL were the major contributors to writing the manuscript. All the authors have read and approved the final version of the manuscript.
