# Supplementary material for: The burden of skin and subcutaneous diseases: findings from the global burden of disease study 2019
Source: Front Public Health. 2023 Apr 17;11:1145513. doi: 10.3389/fpubh.2023.1145513 (PMC10149786; doi:10.3389/fpubh.2023.1145513)
Supplement: Supplementary file 1 [file Data_Sheet_1.pdf]

The Burden of skin and subcutaneous diseases: Findings from the Global Burden of Disease Study 2019

Skin and subcutaneous diseases burden in different countries and territories

At the national level, the highest number of new and deaths cases were recorded in India in 2019, which accounted for 19% of the new cases, and 18% of the death cases detected globally (new case:942,951,782.40 [95% UI, 905,626,668.97–984,569,791.15]; death cases:(18,166.87 [95% UI, 10,388.97–21,877.18]). The highest DALYs, accounting for 19% of the value globally, was recorded in mainland of China in 2019 (8,264,701.92 [95% UI, 5,394,815.62–12,356,265.38]), its new cases number just next to India (784,395,260.74 [95% UI, 819,167,546.48–746,010,720.72]). While Tokelau had the fewest new cases, DALYs and death cases in 2019 (new cases:863.88 [95% UI, 819.83–907.82]); DALYs: (9.37 [95%UI, 6.14–13.70); death cases: (0.03 [95% UI, 0.02-0.04]). Furthermore, we visualized the number of new cases in 2019 among 204 countries and territories by map (Figure S6). From 1990 to 2019, the most significant increase in the number of new cases and DALYs were recorded in Qatar, increased by 566%, and 509%, respectively [In 2019, new cases: 1,180,820.27 (95% UI, 1,131,683.51-1,234,321.48); DALYs: 10,360.70 (95% UI, 6,880.34-15,318.73)]. The most significant reduction of new cases and DALYs were detected in Niue and Georgia, decreasing by 26%, and 37%, respectively [In 2019, new cases 1064.38 (95% UI, 1,010.96–1,114.94); DALYs: 18,493.88 (95% UI, 11,990.43-27,428.21)]. The most significant increase in the number of deaths was detected in the United Arab Emirates, which increased by 736% [In 2019, 13.52 (95% UI, 7.12–25.06)]. The most significant decrease in the number of deaths was detected in Albania, which decreased by 40% [In 2019, 4.61 (95% UI, 3.36–6.92)]. From 1990 to 2019, the type of skin and subcutaneous disease that was the highest contributor to incidence, DALYs, and death cases among 204 countries and territories mostly were fungal skin diseases, dermatitis, and bacterial skin diseases.

In 2019, Ethiopia had the highest ASIR (86,707.16 [95% UI, 81,446.38–92,320.62]), and the lowest ASIR was observed in Greenland (36,303.06 [95% UI, 35,095.76–37,632.65]). France had the highest ASDAR (843.17 [95% UI, 577.86–1,180.26])), while Egypt had the lowest ASDAR (395.58 [95% UI, 263.45–587.03])). Barbados had the highest ASDR (18.17 [95% UI, 10.45–31.01]), whereas Montenegro had the lowest (0.08 [95% UI, 0.06–0.10]) in 2019. From 1990 to 2019, the regions with the largest increase and decrease in the ASIR were Mexico and Poland, increased and decreased by5%, and 4%, respectively [In 2019, Mexico: 67,536.63 (95% UI, 65,345.50–69,893.85); Poland: 46,182.27 (95% UI, 44,022.91–48,583.38)]. As for measures of the ASDAR, the places that exhibited the most significantly increased and decreased DALY values were Saint Vincent and the Grenadines and Bahrain, which increased and decreased by 16%, and 29%, respectively [In 2019, Saint Vincent and the Grenadines: 633.87 (95% UI, 452.92–885.71); Bahrain: 519.06 (95% UI, 377.92–706.79)]. The most significant increase and decrease in the ASDR were detected in Slovakia and Peru, increased

and decreased by 247%, and 68%, respectively [In 2019, Slovakia: 0.35 (95% UI, 0.09–0.46); Peru: 0.54 (95% UI, 0.39–0.72)].

The highest number of new cases and DALYs of acne vulgaris, alopecia areata, dermatitis, pruritus, psoriasis, scabies, Viral skin diseases, and other skin and subcutaneous diseases was recorded in China; The highest number of new cases, of DALYs, death of bacterial skin and subcutaneous diseases, and the highest number of new cases, of DALYs of fungal skin and subcutaneous diseases, urticaria was recorded in India; The highest number of new cases of decubitus ulcers was recorded in the USA from 1990 to 2019, while the highest number of deaths was recorded in the USA from 1990 to 2012, then was recorded in China; The highest DALYs also recorded in the USA from 1990 to 2012 then it recorded China or USA (Figure S2A). The countries or territories that recorded the highest ASIR, ASDAR and ASDR of each skin and subcutaneous disease can be seen in supplementary materials (Figure S2B).11

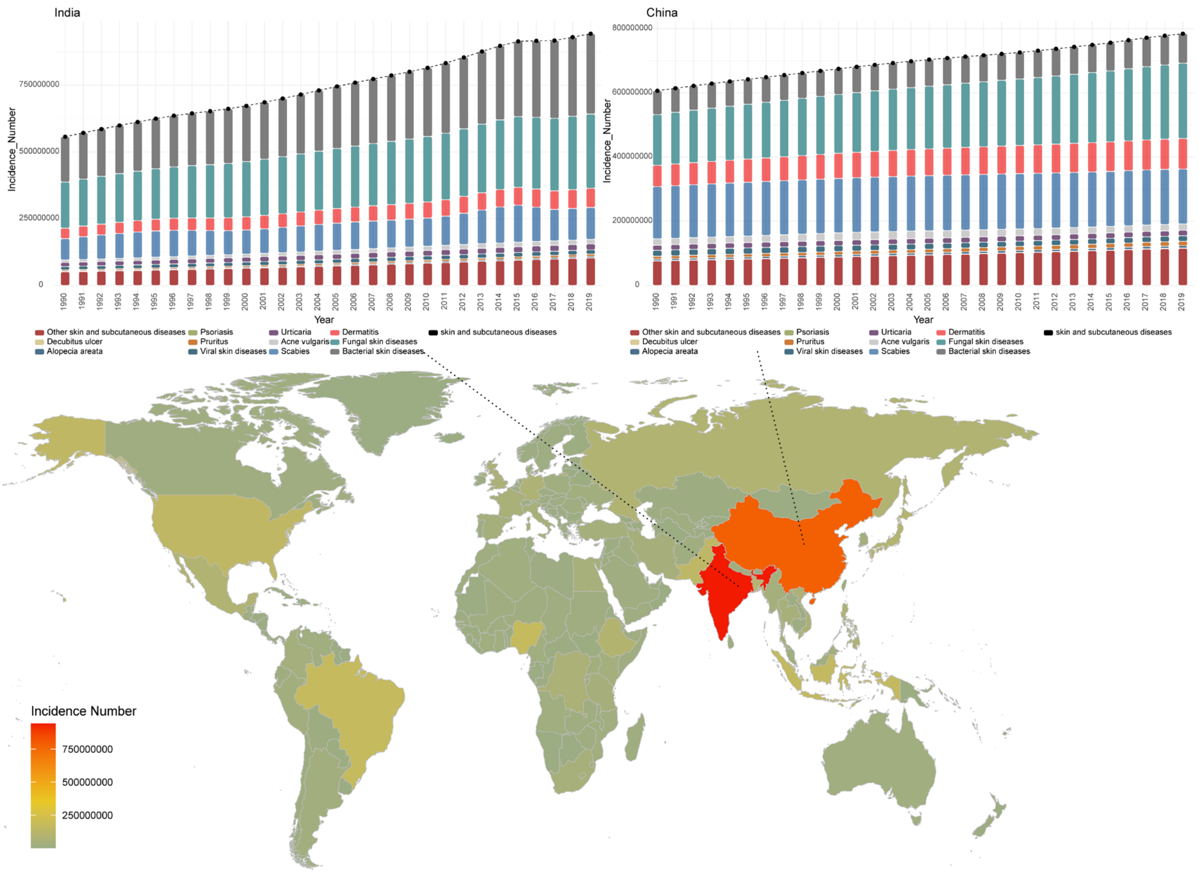

Figure S1. Skin and subcutaneous diseases burden in different countries and territories. the map of new cases in 2019 among 204 countries and territories; the new cases of each skin and subcutaneous disease in India and Mainland China from 1990 to 2019.

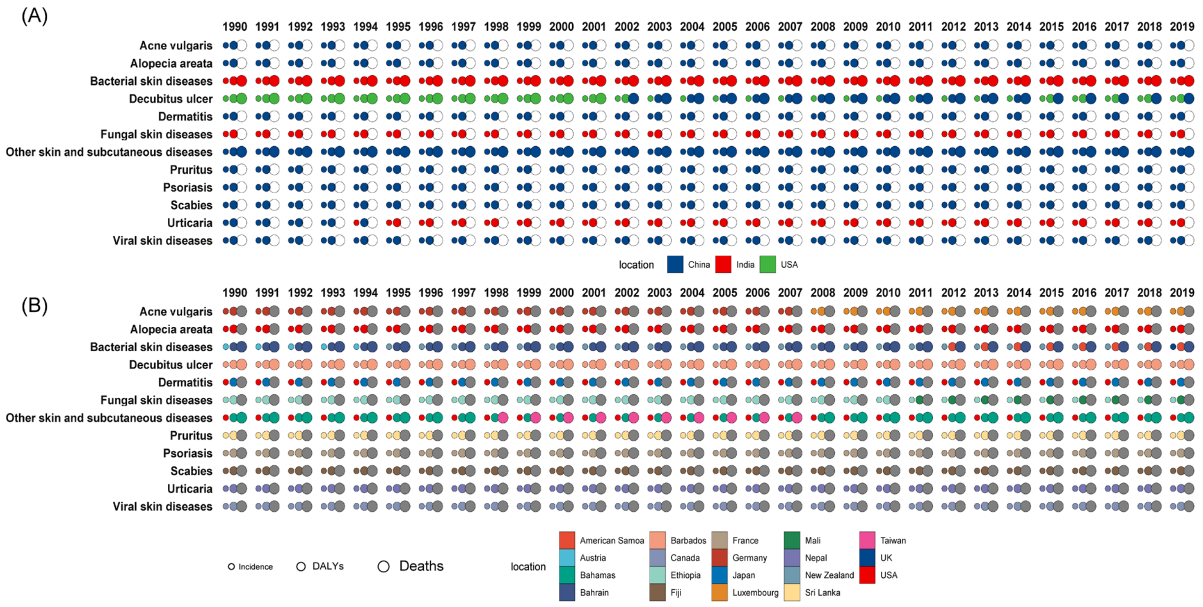

Figure S2. Skin and subcutaneous diseases burden in different countries and territories. (**A**), the countries/territories (204) that recorded the highest new cases, DALYs, and death cases of each skin and subcutaneous disease from 1990 to 2019. (**B**), the countries/territories (204) that recorded the highest ASIR, ASDAR, and ASDR of each skin and subcutaneous disease from 1990 to 2019. ASIR, age-standardized incidence rate; DALYs, disability-adjusted life years; ASDAR, age-standardized DALYs rate; ASDR, age-standardized death rate.

Supplementary Figure legends

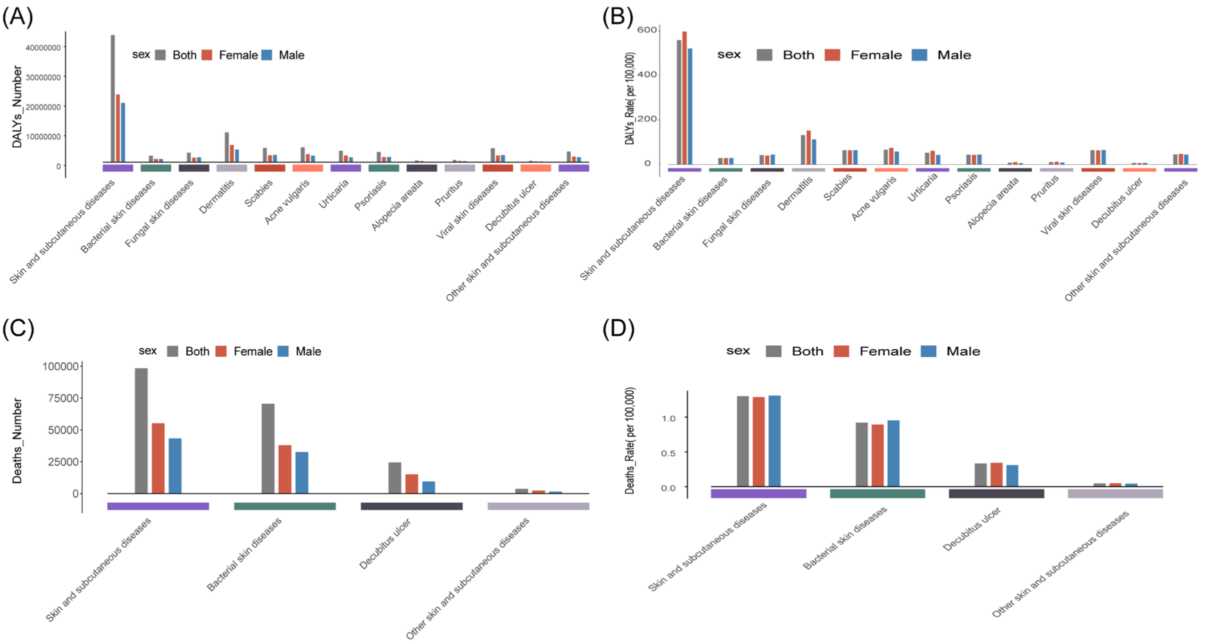

Supplementary Figure S1. Skin and subcutaneous diseases burden among different gender and ages. (**A**), each skin and subcutaneous disease DALYs distribution by sex in 2019. (**B**), each skin and

subcutaneous disease ASDAR distribution by sex in 2019. (**C**), each skin and subcutaneous disease death case distribution by sex in 2019. (**D**), each skin and subcutaneous disease ASDR distribution by sex in 2019. DALYs, disability-adjusted life years; ASDAR, age-standardized DALYs rate; ASDR, age-standardized death rate.

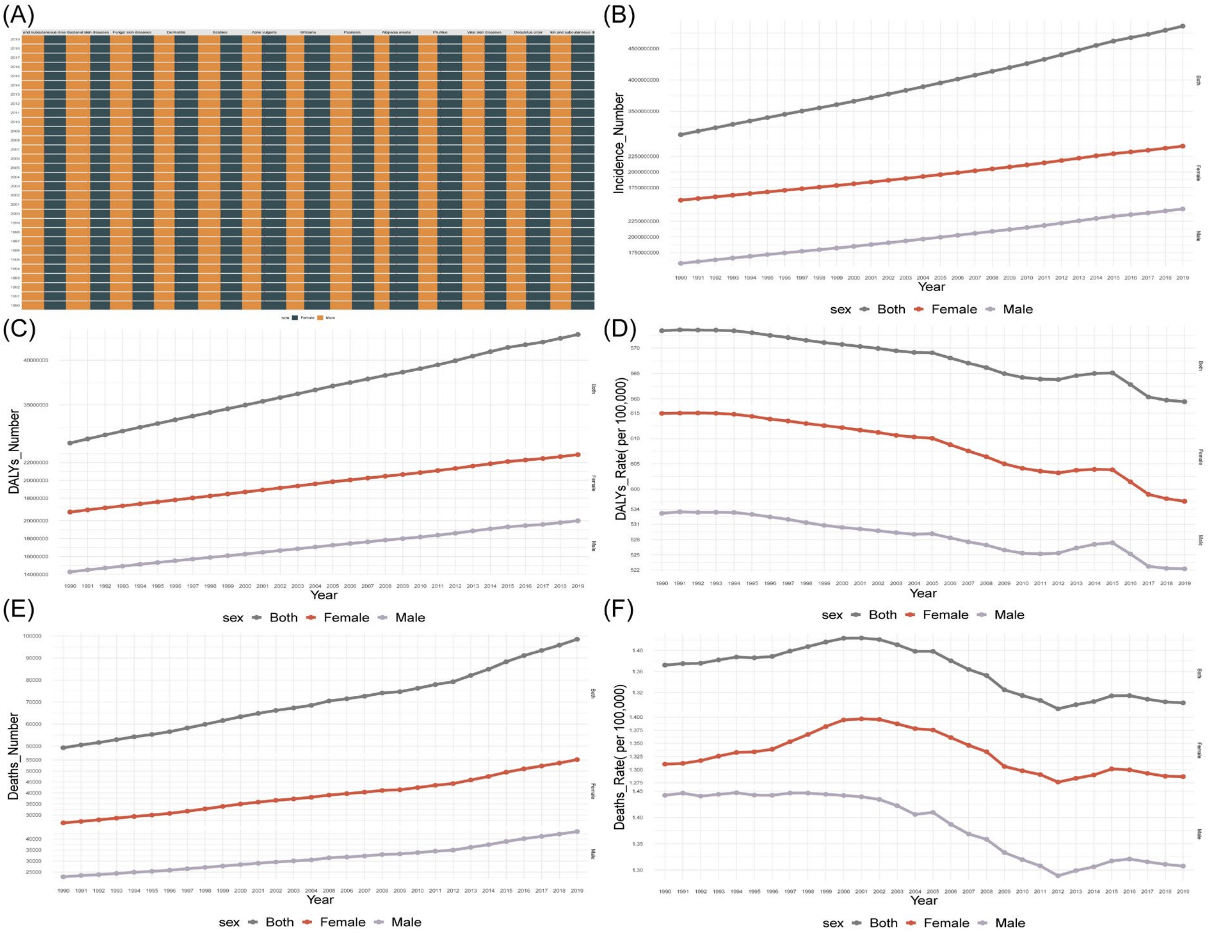

Supplementary Figure S2. Skin and subcutaneous diseases burden among different gender and ages. (**A**), the proportion of males and females in each skin and subcutaneous diseases new cases from 1990 to 2019. (**B**), the global changing trend in each skin and subcutaneous diseases new cases by sex from 1990 to 2019. (**C**), the global changing trend in each skin and subcutaneous disease DALYs by sex from 1990 to 2019. (**D**), the global changing trend in each skin and subcutaneous disease ASDAR by sex from 1990 to 2019. (**E**), the global changing trend in each skin and subcutaneous disease death cases by sex from 1990 to 2019. (**F**), the global changing trend in each skin and subcutaneous disease ASDR by sex from 1990 to 2019. DALYs, disability-adjusted life years; ASDAR, age-standardized DALYs rate; ASDR, age-standardized death rate.

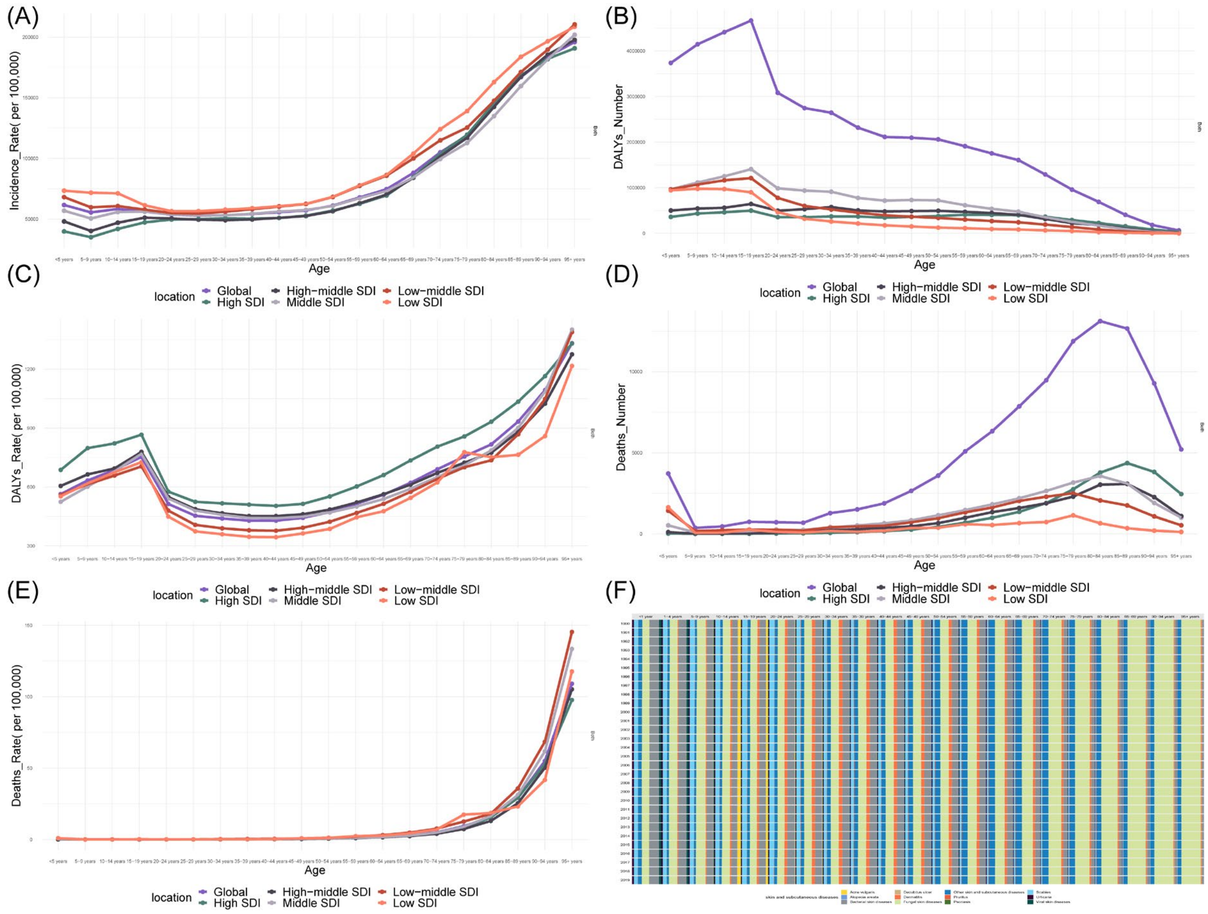

Supplementary Figure S3. Skin and subcutaneous diseases burden among different gender and ages. (**A**), the distribution of all skin and subcutaneous diseases ASIR globally and SDI levels among different age categories in 2019. (**B**), the distribution of all skin and subcutaneous diseases DALYs globally and SDI levels among different age categories in 2019. (**C**), the distribution of all skin and subcutaneous diseases ASDAR globally and SDI levels among different age categories in 2019. (**D**), the distribution of all skin and subcutaneous disease death cases globally and SDI levels among different age categories in 2019. (**E**), the distribution of all skin and subcutaneous diseases ASDR globally and SDI levels among different age categories in 2019. (**F**)**,** the proportion of different age categories in each skin and subcutaneous diseases new cases from 1990 to 2019. ASIR, age-standardized incidence rate; DALYs, disability-adjusted life years; ASDAR, age-standardized DALYs rate; ASDR, age-standardized death rate; SDI, sociodemographic index.

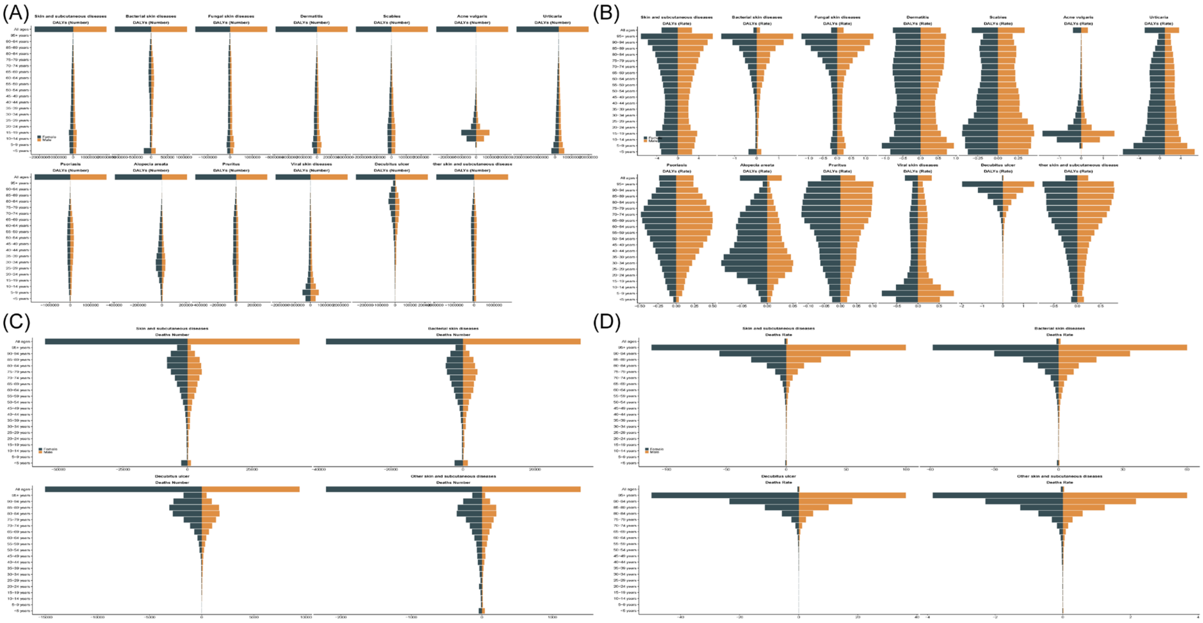

Supplementary Figure S4. Skin and subcutaneous diseases burden among different gender and ages. (**A**), the distribution of each skin and subcutaneous disease DALYs among different age categories in 2019. (**B**), the distribution of each skin and subcutaneous disease DALYs rate among different age categories in 2019. (**C**), the distribution of each skin and subcutaneous disease death cases among different age categories in 2019. (**D**), the distribution of each skin and subcutaneous disease deaths rate among different age categories in 2019. DALYs, disability-adjusted life years.

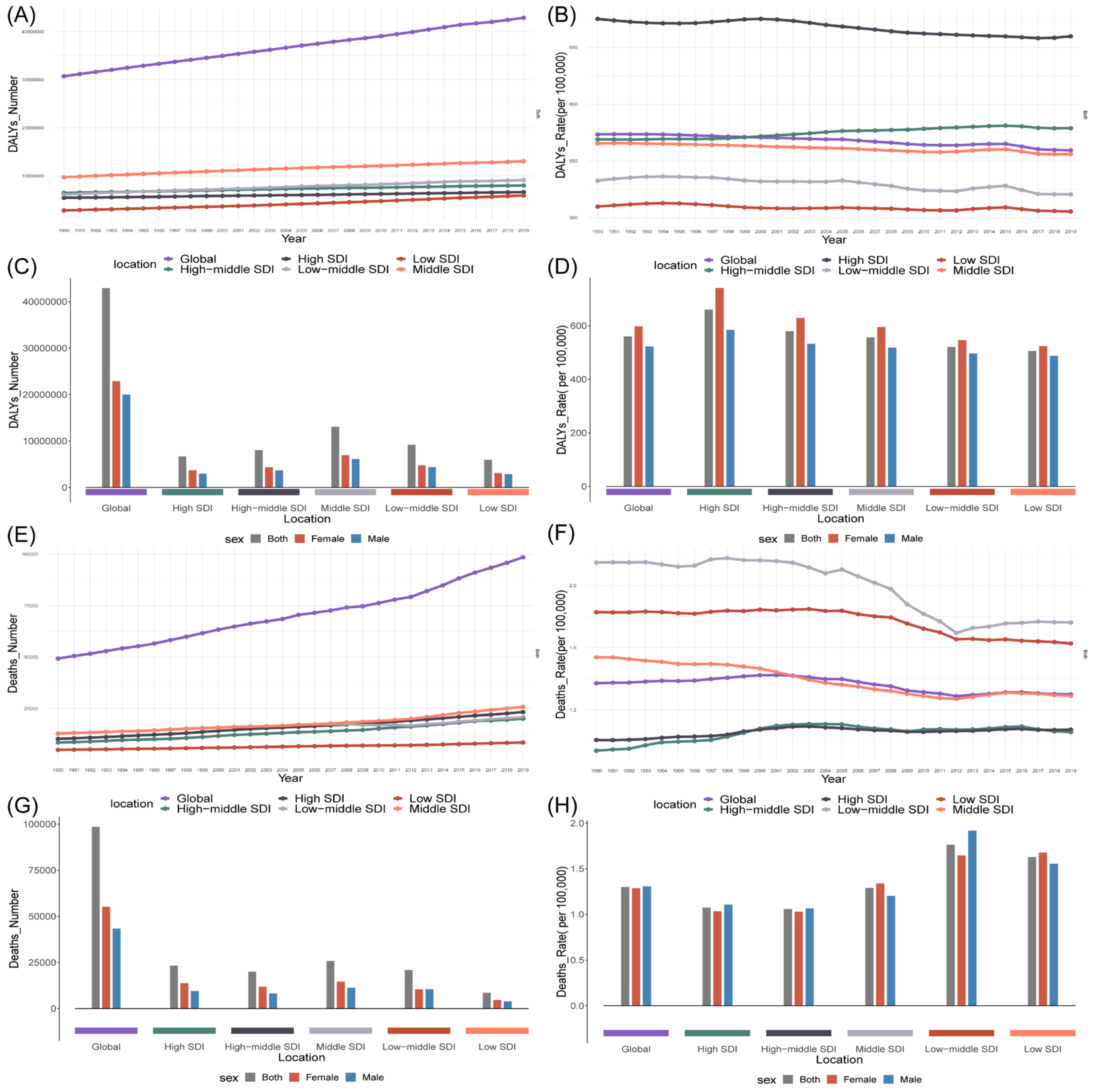

Supplementary Figure S5. Skin and subcutaneous diseases burden among different social-economic states. (**A**), the changing trend in each skin and subcutaneous disease DALYs by global and SDI levels from 1990 to 2019. (**B**), the changing trend in each skin and subcutaneous disease ASDAR by global and SDI levels from 1990 to 2019. (**C**), the skin and subcutaneous diseases DALYs distribution by sex at different SDI levels in 2019. (**D**), the skin and subcutaneous diseases ASDAR distribution by sex at different SDI levels in 2019. (**E**), the changing trend in each skin and subcutaneous disease death cases by global and SDI levels from 1990 to 2019. (**F**), the changing trend in each skin and subcutaneous disease ASDR by global and SDI levels from 1990 to 2019. (**G**), the skin and subcutaneous diseases death cases distribution by sex at different SDI levels in 2019. (**H**), the skin and subcutaneous diseases ASDR distribution by sex at different SDI levels in 2019. DALYs, disability-adjusted life years; ASDAR, age-standardized DALYs rate; ASDR, age-standardized death rate; SDI, sociodemographic index.

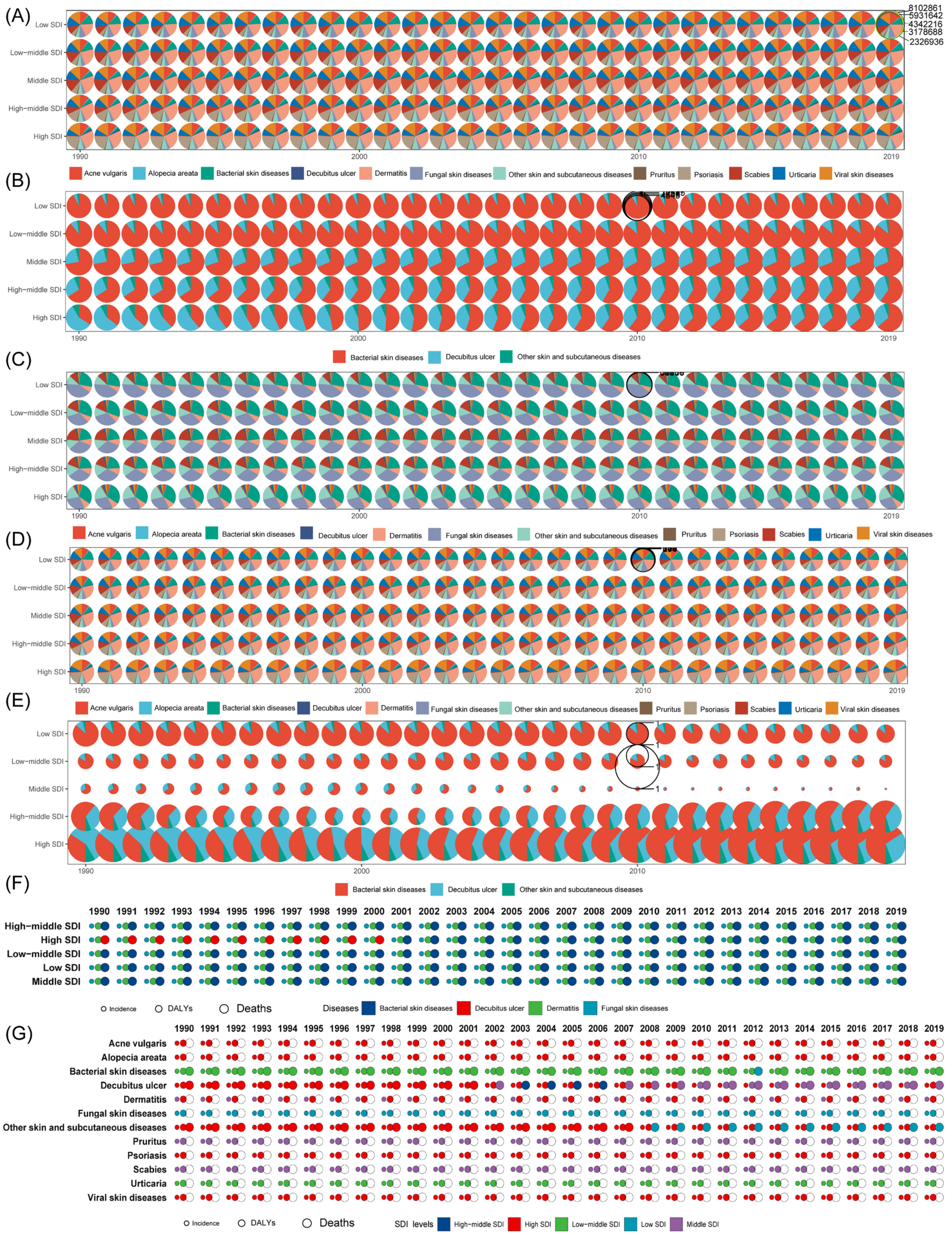

Supplementary Figure S6. Skin and subcutaneous diseases burden in different regions. (**A**), the DALYs proportion of each skin and subcutaneous disease at different SDI levels from 1990 to 2019. (**B**), the death cases proportion of each skin and subcutaneous disease at different SDI levels from 1990 to 2019. (**C**), the ASIR proportion of each skin and subcutaneous disease at different SDI levels from 1990 to 2019. (**D**), the ASDAR proportion of each skin and subcutaneous disease at different SDI levels from 1990 to 2019. (**E**), the ASDR proportion of each skin and subcutaneous disease at different SDI

levels from 1990 to 2019. (**F**), the type of skin and subcutaneous diseases that contribute the highest ASIR, ASDAR, and ASDR at different SDI levels from 1990 to 2019. (**G**), the SDI levels that recorded the highest ASIR, ASDAR, and ASDR of each skin and subcutaneous disease from 1990 to 2019. GBD, Global Burden of Disease; ASIR, age-standardized incidence rate; DALYs, disability-adjusted life years; ASDAR, age-standardized DALYs rate; ASDR, age-standardized death rate; SDI, sociodemographic index.

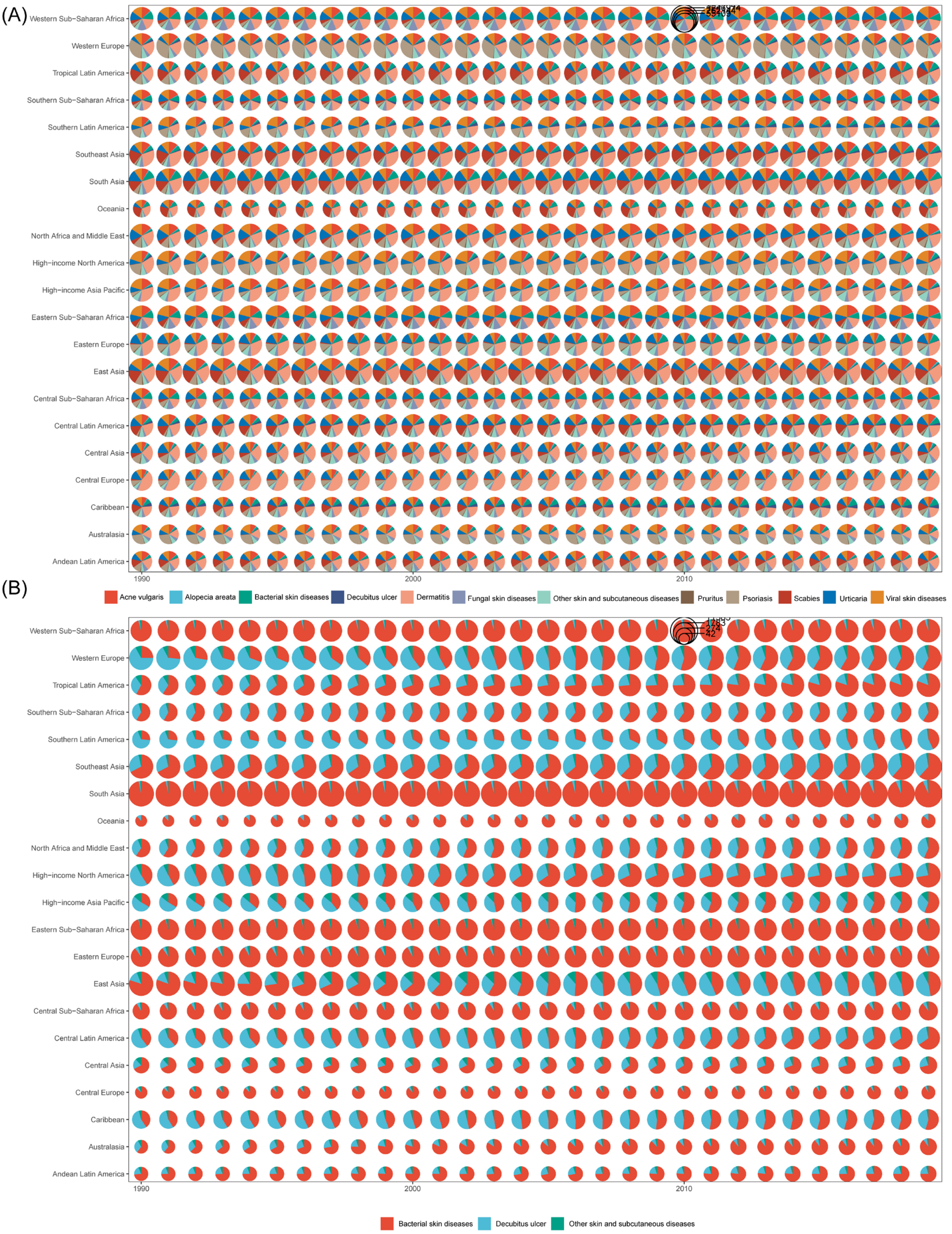

Supplementary Figure S7. Skin and subcutaneous diseases burden in different regions. (A), the DALYs proportion of each skin and subcutaneous disease at different GBD regions (21) from 1990 to 2019. (B), the death cases proportion of each skin and subcutaneous disease at different GBD regions (21) from 1990 to 2019. GBD, Global Burden of Disease; DALYs, disability-adjusted life years.

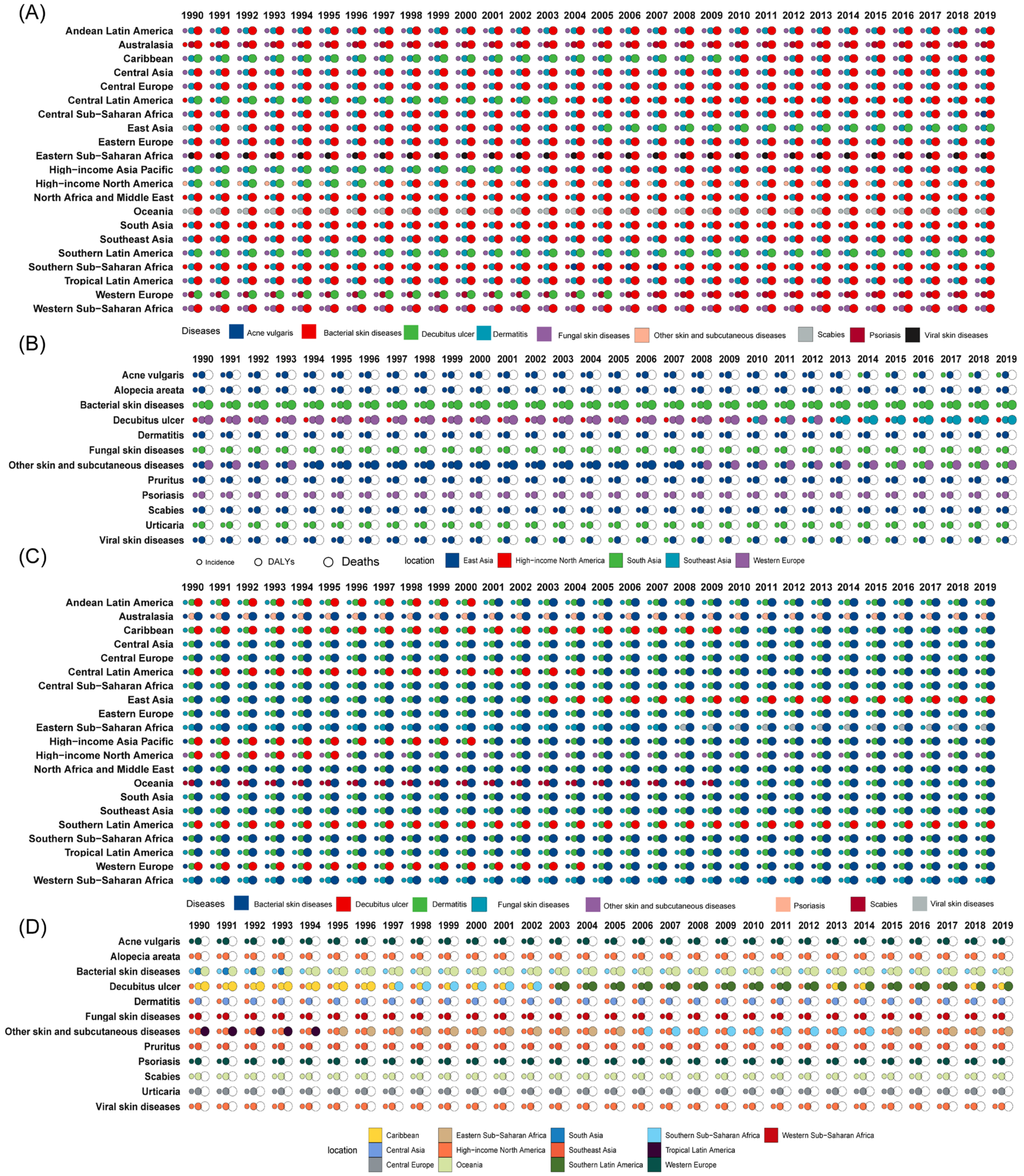

Supplementary Figure S8. Skin and subcutaneous diseases burden in different regions. (**A**), the type of skin and subcutaneous diseases that contribute the highest new cases, DALYs, and death cases at different GBD regions (21) from 1990 to 2019. (**B**), the GBD regions (21) that recorded the highest new cases, DALYs, and death cases of each skin and subcutaneous disease from 1990 to 2019. (**C**), the type of skin and subcutaneous diseases that contribute the highest ASIR, ASDAR, and ASDR at different GBD regions (21) from 1990 to 2019. (**D**), the GBD regions (21) that recorded the highest ASIR, ASDAR, and ASDR of each skin and subcutaneous disease from 1990 to 2019. GBD, Global Burden of Disease; ASIR, age-standardized incidence rate; DALYs, disability-adjusted life years; ASDAR, age-standardized DALYs rate; ASDR, age-standardized death rate.

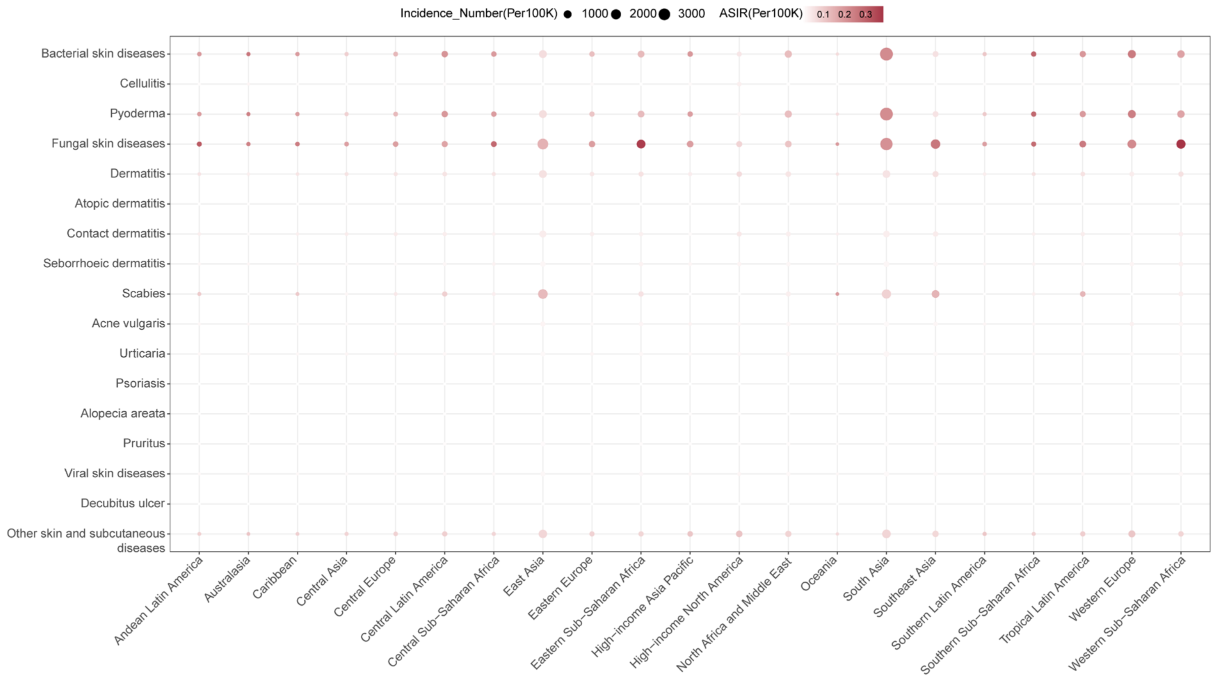

Supplementary Figure S9. The comparison of skin and subcutaneous disease burden in different regions. the comparison of each skin and subcutaneous diseases burden at different GBD regions (21) by their new cases and ASIR in 2019. GBD, Global Burden of Disease; ASIR, age-standardized incidence rate.

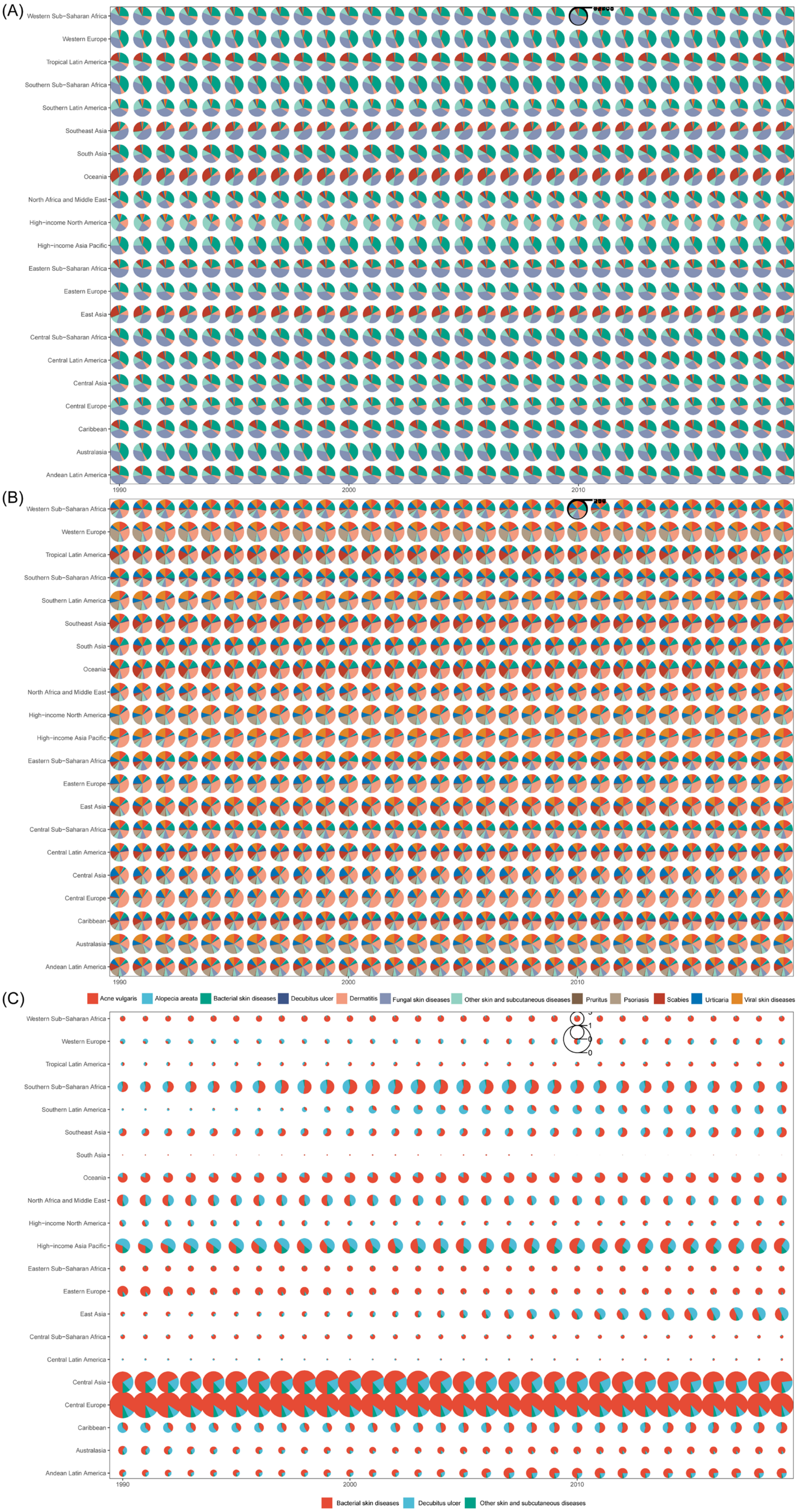
(A)
(B)
(C)
Western Sub-Saharan Africa
Western Europe
Tropical Latin America
Southern Sub-Saharan Africa
Southern Latin America
Southeast Asia
South Asia
Oceania
North Africa and Middle East
High-income North America
High-income Asia Pacific
Eastern Sub-Saharan Africa
Eastern Europe
East Asia
Central Sub-Saharan Africa
Central Latin America
Central Asia
Central Europe
Caribbean
Australasia
Andean Latin America
1990
2000
2010
Acne vulgaris
Alopecia areata
Bacterial skin diseases
Decubitus ulcer
Dermatitis
Fungal skin diseases
Other skin and subcutaneous diseases
Pruritus
Psoriasis
Scabies
Urticaria
Viral skin diseases
Bacterial skin diseases
Decubitus ulcer
Other skin and subcutaneous diseases

Supplementary Figure S10. Skin and subcutaneous diseases burden in different regions. (**A**), the ASIR proportion of each skin and subcutaneous disease at different GBD regions (21) from 1990 to 2019. (**B**), the ASDAR proportion of each skin and subcutaneous disease at different GBD regions (21) from 1990 to 2019. (**C**), the ASDR proportion of each skin and subcutaneous disease at different GBD regions (21) from 1990 to 2019. GBD, Global Burden of Disease; ASIR, age-standardized incidence rate; ASDAR, age-standardized DALYs rate; ASDR, age-standardized death rate.

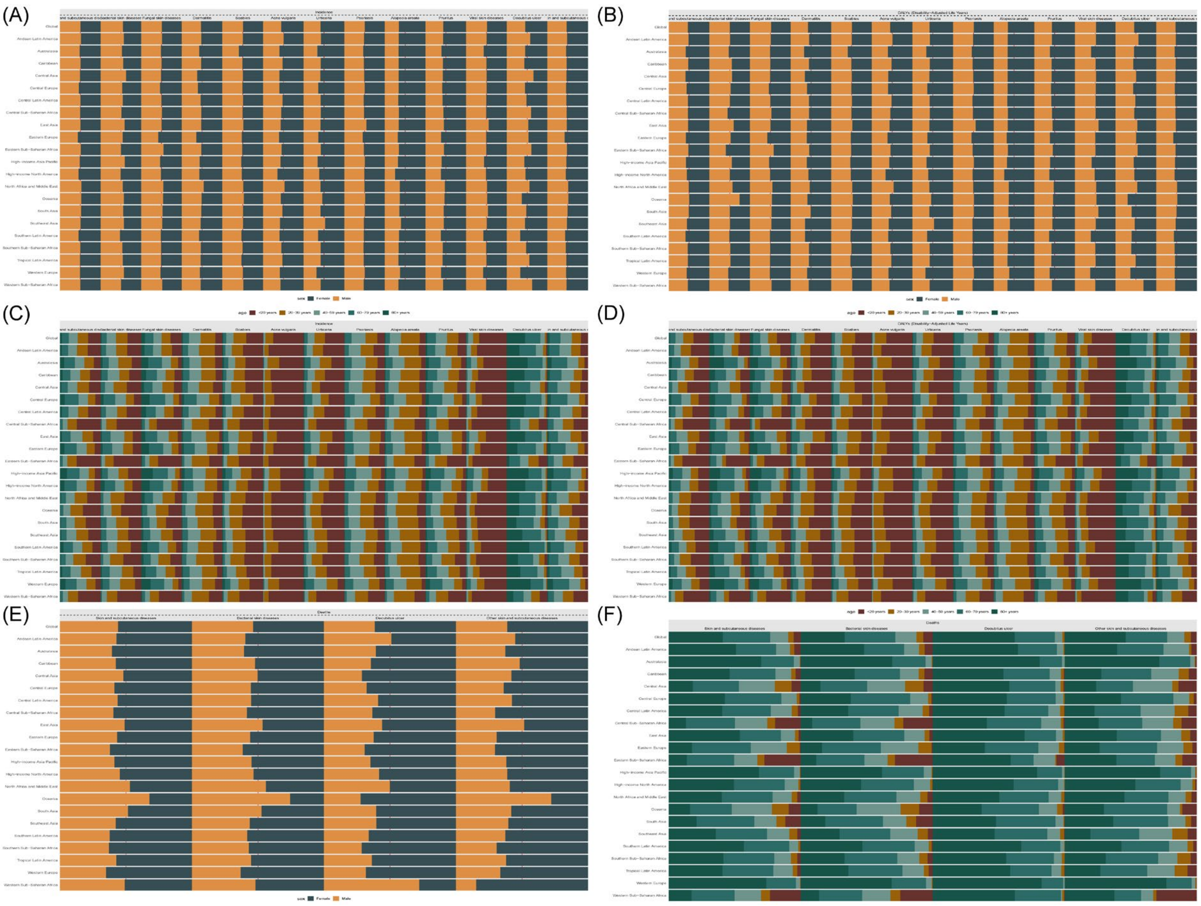

Supplementary Figure S11. Skin and subcutaneous diseases burden in different regions. (**A**), the proportion of males and females in each skin and subcutaneous diseases new cases at different GBD regions (21) in 2019. (**B**), the proportion of males and females in each skin and subcutaneous diseases DALYs at different GBD regions (21) in 2019. (**C**), the proportion of different age categories in each skin and subcutaneous diseases new cases at different GBD regions (21) in 2019. (**D**, the proportion of different age categories in each skin and subcutaneous disease DALYs at different GBD regions (21) in 2019. (**E**), the proportion of males and females in each skin and subcutaneous disease death cases at different GBD regions (21) in 2019. (**F**), the proportion of different age categories in each skin and subcutaneous disease death cases at different GBD regions (21) in 2019. GBD, Global Burden of Disease; DALYs, disability-adjusted life years.

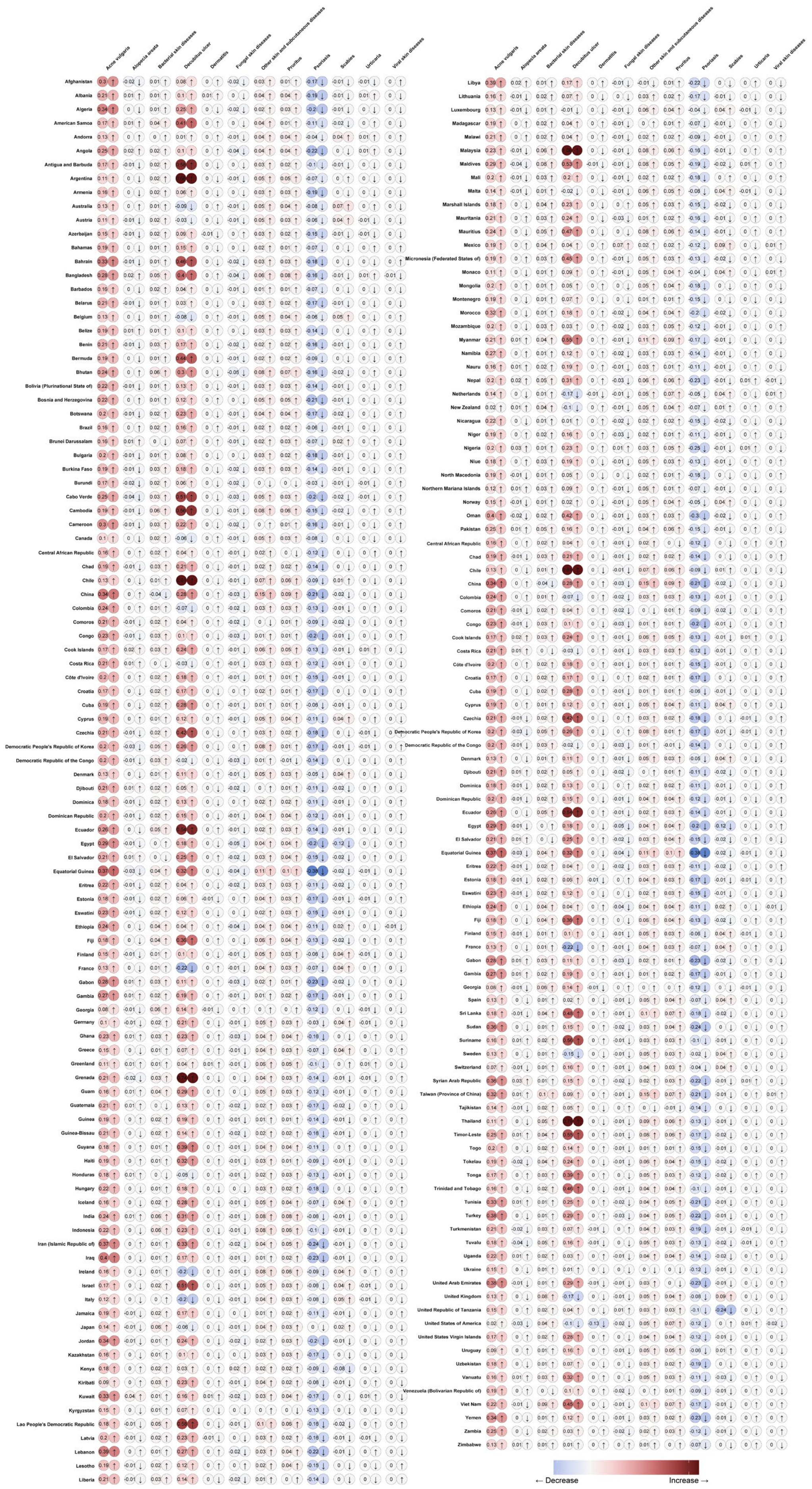
Acne vulgaris
Alopecia areata
Bacterial skin diseases
Decubitus ulcer
Dermatitis
Fungal skin diseases
Other skin and subcutaneous diseases
Pruritus
Psoriasis
Scabies
Urticaria
Viral skin diseases
← Decrease
Increase →

Supplementary Figure S12. The trend of skin and subcutaneous diseases. the trends of each skin and subcutaneous disease among 204 countries and territories.

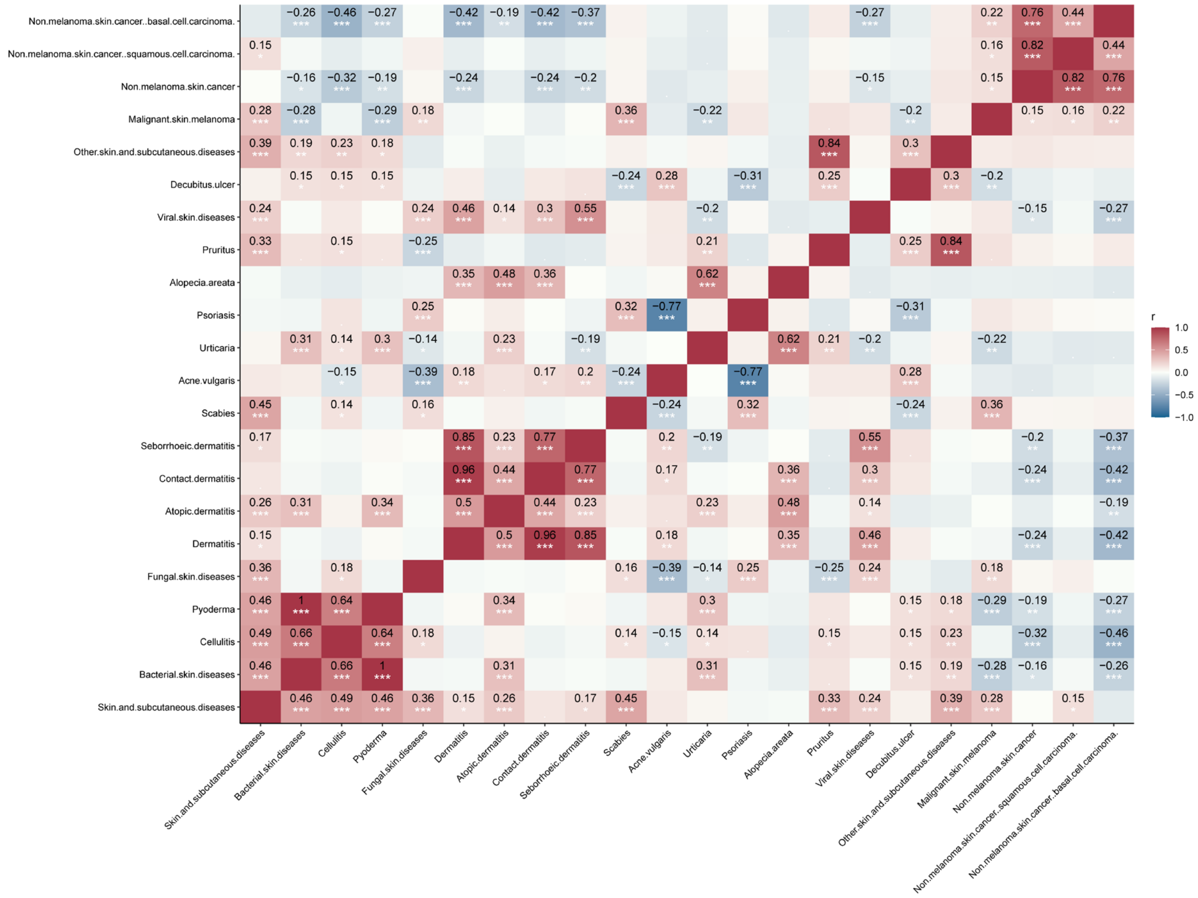

Supplementary Figure S13. The relevance between the skin and subcutaneous diseases and skin cancer. the correlation analysis between each skin and subcutaneous disease and skin cancer's annual age-standardized incidence rate of changes.

The Burden of skin and subcutaneous diseases: Findings from the Global Burden of Disease Study 2019

Supplementary Table 1. The number of new cases of skin and subcutaneous diseases at Global, Regional, and National levels in 2019.

	No. (95% UI)							
	2019							
**Diseases**	Global Incident cases	Percent	21 GBD region	new cases	Percent	Nation	new cases	Percent
**skin and subcutaneous diseases**	4859267653.45 (4680693439.81 to 5060498767.38)	-	South Asia	1208952415 (1162257056 to 1259536849)	24.88%	India	942951782.398206 (905626668.966967 to 984569791.154241)	19.41%
**Bacterial skin and subcutaneous diseases**	1134138000.37 (1105834933.32 to 1169419691.06)	23.34%	South Asia	389782349.3 (105580259 to 111445960.6)	34.37%	India	300985253.1 (7782794.837 to 8300087.705)	26.54%
**Cellulitis**	42909913.36 (40371149.36 to 45427001.71)	0.88%	High-income North America	12340047.5442832 (11726899.0969036 to 12965513.6298876)	1.09%	India	5458633.78880761 (5095643.97024499 to 5840600.76742514)	0.48%
**Pyoderma**	1091228087 (1062900135.16 to 1125735702.6)	22.46%	South Asia	382830278.405286 (373037485.897019 to 395381433.338936)	33.76%	India	295526619.293634 (287947314.222378 to 305327206.820342)	26.06%
**Fungal skin and subcutaneous diseases**	1646596956.43 (1493240310.67 to 1811901501.32)	33.89%	South Asia	347519523.5 (49374064.41 to 62698306.97)	21.11%	India	278295577.5 (549963.5047 to 692044.0849)	16.90%
**Scabies**	565054267.35 (498694655.46 to 634038367.27)	11.63%	East Asia	176585120.5 (1842200.646 to 2306189.44)	31.25%	China	170879002.7 (96561.76995 to 122398.4178)	30.24%
**Dermatitis**	418444538.24 (361104784.53 to 477975663.91)	8.61%	East Asia	97923270.52 (5744236.098 to 8246193.46)	23.40%	China	94910074.42 (146889.6059 to 203226.9688)	22.68%
**Atopic dermatitis**	24446018.31 (23339681.97 to 25569146.46)	0.50%	East Asia	6080367.18999781 (5807760.67030006 to 6330811.25657429)	1.45%	China	5837355.40326893 (5576377.22897548 to 6077542.15182946)	1.40%
**Contact dermatitis**	249404725.28 (194965911.34 to 305455885.67)	5.13%	East Asia	65733253.7308976 (50858462.7534355 to 81466122.2214125)	15.71%	China	63827548.2255342 (49397429.3107181 to 79156504.3738475)	15.25%
**Seborrhoeic dermatitis**	144593794.64 (133314560.37 to	2.98%	South Asia	32636378.3159594 (29989840.8575212 to	7.80%	China	25245170.789084 (23187400.1613653 to	6.03%

	155584506.36)			35403722.0694163)			27321425.3432066)	
**Acne vulgaris**	117366804.28 (102959354.62 to 133667283.46)	2.42%	South Asia	22003983.92 (2622700.172 to 3258311.036)	18.75%	China	20197656.26 (18098.89563 to 22465.9466)	17.21%
**Urticaria**	114708912.18 (101309687.09 to 129286333.7)	2.36%	South Asia	31399687.34 (1973191.733 to 2445545.958)	27.37%	India	22065409.43 (42760.02791 to 54420.10087)	19.24%
**Viral skin diseases**	98949562.66 (95060186.54 to 102759028.76)	2.04%	South Asia	21246358.92 (2158841.677 to 2292464.248)	21.47%	China	17144833.22 (22372.91279 to 24606.29371)	17.33%
**Pruritus**	57008020.37 (50729476.86 to 63641352.62)	1.17%	East Asia	14354978.15 (489772.8445 to 617810.0566)	25.18%	China	13899034.2 (17744.12927 to 22593.90091)	24.38%
**Alopecia areata**	32426829.18 (31370861.58 to 33473493.05)	0.67%	East Asia	6898387.728 (282422.1045 to 304892.321)	21.27%	China	6667732.762 (10611.71489 to 11374.01142)	20.56%
**Psoriasis**	4622593.7 (4458903.99 to 4780771.12)	0.10%	Western Europe	946916.1834 (74395.75382 to 79469.46119)	20.48%	China	892905.9438 (1742.736099 to 1884.492929)	19.32%
**Decubitus ulcer**	3170795.93 (2875432.71 to 3499728.67)	0.07%	High-income North America	819080.0686 (199723.7781 to 258461.4161)	25.83%	USA	744426.1329 (1137.824826 to 1464.780301)	23.48%
**Other skin and subcutaneous diseases**	666780372.76 (652013798.2 to 683098895.8)	13.72%	South Asia	130819241.1 (29786962.98 to 31333329.12)	19.62%	China	115715632.1 (266331.5349 to 284909.7175)	17.35%

Abbreviations: UI, uncertainty interval.

Supplementary Table 2. The Deaths Cases of skin and subcutaneous diseases at Global, Regional, and National levels in 2019.

	No. (95% UI)							
	2019							
Diseases	Global Death cases	Percent	21 GBD region	Death cases	Percent	Nation	Deaths cases	Percent
**Bacterial skin and subcutaneous diseases**	70526.47 (51665.19 to 87438.6)	71.58%	South Asia	17756.6948443928 (3626.77391460164 to 2411.65387403951)	25.18%	India	16549.43（9782.51 to 20018.79）	23.47%
**Cellulitis**	18068.54 (11393.79 to 23721.19)	18.34%	High-income North America	3448.10054164479 (1167.46531863921 to 215.349594544003)	19.08%	United States of America	3135.15（1665.32 to 4885.11）	17.35%
**Decubitus**	24388.57 (17299.07	24.75%	Southeast Asia	5073.37092254736	20.80%	China	2686.99（1960.62 to 3269.02）	11.02%

**ulcer**	to 31260.82)			(64.924428002754 to 24.9719043333959)				
**Other skin and subcutaneous diseases**	3606.63 (2684.35 to 5022.35)	3.66%	Western Europe	651.743127544932 (200.259159483987 to 117.422318601219)	18.07%	China	414.51（333.47 to 604.85）	11.49%
**skin and subcutaneous diseases**	98521.67(123948.5 6 to 75116.11)	100.00%	South Asia	19520.8785051733 (3868.16887604127 to 2589.99928906267)	19.81%	India	18166.87（10388.97 to 21877.18）	18.44%
**Pyoderma**	52457.93 (38072.96 to 64961.44)	53.25%	South Asia	15998.225626131 (2985.52997410225 to 1789.15972849256)	30.50%	India	14879（8441.57 to 18405.2）	28.36%

Abbreviations: UI, uncertainty interval.

Supplementary Table 3. The DALYs of skin and subcutaneous diseases at Global, Regional, and National level in 2019.

	No. (95% UI)							
	2019							
**Diseases**	Global DALYs	Percent	21 GBD region	DALYs	Percent	Nation	DALYs	Percent
**Acne vulgaris**	4955197.67 (2978062.72 to 7853723.69)	11.55%	East Asia	953896.465157463 (27856.4326279523 to 74811.8983030516)	19.25%	China	921792.84 (555005.72 to 1462139.58)	18.60%
**Alopecia areata**	600570.37 (378239.38 to 891060.98)	1.40%	East Asia	129127.75646084 (2913.16478920743 to 6783.38732282379)	21.50%	China	124821.98 (78771.44 to 185268.44)	20.78%
**Atopic dermatitis**	7480085.12 (3986820.73 to 12581232.09)	17.44%	East Asia	1608174.1924748 (151339.835244715 to 465931.61170803)	21.50%	China	1539726.9 (824236.23 to 2559834.38)	20.58%
**Bacterial skin and subcutaneous diseases**	2197262.6 (1656534.08 to 2639494.59)	5.12%	South Asia	686784.014617183 (7123.31531232859 to 12489.6415474086)	31.26%	India	635992.53 (374327.07 to 781709.19)	28.94%
**Cellulitis**	551446.01 (377644.49 to 660369.44)	1.29%	South Asia	94490.4187520792 (1487.40263440496 to 2580.21376505641)	17.14%	India	87414.63 (52865.22 to 117861.58)	15.85%
**Contact dermatitis**	2275936.53 (1431634.67 to 3396560.78)	5.31%	East Asia	592949.874813213 (13788.8481177914 to 33490.4138277151)	26.05%	China	575659.91 (358389.38 to 869506.14)	25.29%
**Decubitus ulcer**	481422.76 (374333.94 to 583429.14)	1.12%	Southeast Asia	95781.1394489155 (1379.12805390742 to 2289.7819135681)	19.90%	United States of America	52227.89 (39828.6 to 72650.8)	10.85%
**Dermatitis**	10063791.45 (5956892.77 to	23.47%	East Asia	2256559.36538209 (44029.7390764057 to	22.42%	China	2168947.04 (1278715.98 to 3359954.47)	21.55%

	15750929.61)			115932.035700892)				
**Fungal skin and subcutaneous diseases**	3224429.95 (1322041.16 to 6784674.96)	7.52%	South Asia	658358.168119234 (17618.5388314516 to 94650.7876922376)	20.42%	India	533438.98 (217793.27 to 1083752.62)	16.54%
**Other skin and subcutaneous diseases**	3604631.05 (1773654.95 to 6498917.07)	8.41%	South Asia	693917.471174454 (13788.7930630944 to 50978.3665092808)	19.25%	China	636795.91 (310215.4 to 1150821.76)	17.67%
**Pruritus**	785750.47 (376081.72 to 1405750.85)	1.83%	East Asia	199876.223163295 (2515.49076885205 to 9391.46289134506)	25.44%	China	193515.68 (91589.62 to 347593.01)	24.63%
**Psoriasis**	3505736.13 (2504955.77 to 4638756.5)	8.17%	Western Europe	871673.266899269 (27361.5925591957 to 51289.879115459)	24.86%	China	662715.26 (471944.22 to 875803.12)	18.90%
**Pyoderma**	1645816.59 (1245783.54 to 2008094.98)	3.84%	South Asia	592293.595865105 (5354.57636994727 to 10117.4528911594)	35.99%	India	548577.9 (322829.95 to 688959.02)	33.33%
**Scabies**	4837980.63 (2682199.12 to 7727672.44)	11.28%	East Asia	1511501.99813225 (28936.7331691403 to 83812.2484066221)	31.24%	China	1462741.24 (811707.48 to 2338050.58)	30.23%
**Seborrhoeic dermatitis**	307769.79 (175925.71 to 489314.82)	0.72%	South Asia	65592.190622996 (1190.13105476154 to 3379.05372595424)	21.31%	China	53560.23 (30447.71 to 85707.53)	17.40%
**skin and subcutaneous diseases**	42883695.48 (63438210.22 to 28626691.71)	100.00%	South Asia	8965385.4462264 (241558.439017456 to 563335.963377922)	20.91%	China	8264701.92 (5394815.62 to 12356265.38)	19.27%
**Urticaria**	4728083.85 (3026072.11 to 7062875.76)	9.09%	South Asia	1068940.87798163 (19985.076658849 to 44502.1938633016)	27.42%	India	749495.88 (490301.26 to 1074876.01)	19.22%
**Viral skin diseases**	4955197.67 (2978062.72 to 7853723.69)	11.03%	East Asia	1036694.00724217 (21141.654610485 to 50095.3508226465)	21.93%	China	996451.86 (635427.51 to 1475630.57)	21.08%

Abbreviations: UI, uncertainty interval.

Supplementary Table 4. The burden of each skin and subcutaneous diseases among sex.

	No. (95% UI)

		1990						2019					
Diseases	Sex	New cases	DALYs	Death cases	ASIR	ASDAR	ASDR	New cases	DALYs	Death cases	ASIR	ASDAR	ASDR
**Acne vulgaris**	Female	45201264.2884517 (39731111.7785293 to 51469049.2070021)	1908963.29950017 (1150869.96406837 to 3027328.90900857)		1559.05302180194 (1373.65739419357 to 1774.94430658971)	65.6199832607552 (39.438514300226 to 103.836816813911)		64961744.2120051 (57163079.953199 to 73689296.672613)	2285813.6664272 (1470710.38698538 to 3419393.51773688)		1796.02779376913 (1570.81553041576 to 2045.93618883962)	74.4685528636113 (44.8372238397907 to 117.90921724404)	
	Male	34522266.0008961 (30189852.9494086 to 39449330.9106059)	1447598.1523085 (870185.338657138 to 2302130.70785236)		1141.05761698999 (1000.55159905144 to 1301.60205085629)	47.7923215600671 (28.736148698275 to 75.8889083873752)		52405060.0718909 (45855432.5630584 to 59924737.0681318)	2442270.17955953 (1554992.87805001 to 3641922.9171563)		1384.94292345351 (1206.66580004984 to 1586.90383142926)	57.6347084177581 (34.4332796007163 to 91.578771909722)	
**Alopecia areata**	Female	14120922.2607884 (13646428.925712 to 14606053.3447766)	259562.912027204 (162753.617604571 to 384654.864828672)		549.025553900425 (531.314886413424 to 567.348870196789)	10.0988377335303 (6.3521276657577 to 15.0088896099333)		21267565.5364683 (20564412.8319959 to 21965028.7698609)	2285568.79162961 (1499168.11541432 to 3272442.290764)		531.682471275599 (514.174794532128 to 548.869147189049)	9.79972096338913 (6.17988656176173 to 14.5113511486662)	
	Male	7621914.18647627 (7335584.38670079 to 7888360.06954396)	142119.25203638 (88933.1467795993 to 210937.281229553)		287.259754763789 (277.016964971259 to 296.784701033727)	5.36159664091297 (3.36082402406752 to 7.96962804873153)		11159263.6446383 (10773816.9759711 to 11522569.7051642)	1613269.76433068 (1051942.79045795 to 2320511.35037128)		279.176591928357 (269.584770910412 to 288.254419417836)	5.215631832625 (3.26614332452194 to 7.78909624034095)	
**Atopic dermatitis**	Female	11442757.1416186 (10897505.8237087 to 12006282.1688034)	3406120.75175709 (1806128.11459528 to 5706097.98464477)		413.812015952193 (394.90240057951 to 433.174811530781)	123.726722839247 (65.7920905667559 to 207.27889309155)		14513119.1116902 (13846232.6348157 to 15151479.2201285)	22881271.1721675 (15314891.9298582 to 33748604.8772621)		390.541977626685 (372.4707441726 to 408.321847066983)	117.651908133315 (62.6066758828256 to 197.415047860333)	
	M	7744	2421		272.3	84.58		9932	2000		266.6	82.01	

	ale	403.55725047 (7352464.49660076 to 8179751.33530821)	021.26594159 (1287579.62667369 to 4067473.39762827)		40022988234 (259.003064266354 to 286.100563295368)	91111164098 (45.1163463045008 to 142.106614832083)		899.20138694 (9452870.38793199 to 10421103.7689898)	2424.3077692 (13364228.3836715 to 29689605.342491)		89423859248 (253.841980546935 to 280.249214805655)	08795238979 (43.7199905255857 to 137.718604983184)	
Bacterial skin diseases	Female	326922525.611936 (318394396.538891 to 337337319.380133)	805220.612712632 (518372.626133311 to 1068214.32277118)	17342.0849842511 (12004.7670219461 to 22688.7657224882)	12278.1351440191 (11975.3069187156 to 12648.5940013414)	30.7140204450372 (20.7478285003543 to 39.9499490007424)	0.79084419765988 (0.563784637475998 to 1.02773520396393)	509583854.633637 (497397624.029867 to 524976276.867395)	152158.915883175 (86662.2229652351 to 241541.513496686)	37923.5764485322 (28296.1394025967 to 48862.2826763715)	13354.014760263 (13024.4413572921 to 13758.4942166141)	28.1188107009357 (22.0491314179436 to 33.9075231253474)	0.892998774608622 (0.667377524634024 to 1.14425859432595)
	Male	400551242.709615 (389773603.8764 to 413404016.234923)	800954.259811923 (471305.212286226 to 1047673.91204225)	17147.5071392567 (9908.50382105304 to 22105.7381775758)	15091.6242177621 (14713.7073030251 to 15561.1203925928)	33.0225828402676 (20.5160238146552 to 42.2396699371822)	0.969088626365616 (0.573291953366892 to 1.23748716428927)	624554145.734859 (608812820.274895 to 643962236.81431)	155610.873314758 (88185.1971925276 to 248695.427563678)	32602.8905125297 (19170.741281702 to 45168.9929130638)	16048.4064874815 (15644.7186849567 to 16545.0833908983)	28.8288422271479 (18.8282950133913 to 36.6850111229673)	0.953094212963553 (0.56299200513644 to 1.34306995740468)
Cellulitis	Female	13406621.9957283 (12562579.6375879 to 14230667.4189046)	188512.775312727 (107031.686654216 to 315502.320993761)	4268.38235709511 (2739.51038097937 to 6582.768529863)	540.66413018682 (508.392894220684 to 572.874286612658)	7.48485953435618 (4.55384599648958 to 11.9855188077142)	0.19930183160566 (0.133341340019204 to 0.299705742069138)	20153832.9005422 (18989697.0619668 to 21303054.0039626)	2382486.21452146 (1320585.12669179 to 3810800.16272659)	9753.73022460291 (5451.19451359884 to 13813.9951055592)	507.91335781416 (478.072611688549 to 536.890182559175)	6.70498508150976 (4.18015155610354 to 8.12712872488308)	0.226591344208839 (0.126895575502191 to 0.317980377493059)
	Male	15388936.7262091 (14359496.6940533 to 16352762.1934306)	193191.175863536 (110909.436675289 to 293764.654456986)	4036.69115899649 (2112.7116471394 to 6966.23606652485)	624.542980647134 (586.792480940657 to 662.329607886456)	8.1026090763684 (4.83207156147297 to 12.4116585283243)	0.224972945326534 (0.122029151720228 to 0.401063125383691)	22756080.4637932 (21394825.2359301 to 24123078.7734182)	2455494.41284013 (1356481.36407177 to 3917174.18892029)	8314.80556239396 (3707.17878834124 to 13252.5399055611)	588.920412507666 (554.256430440511 to 623.469424255993)	7.22485338290745 (4.02954750266241 to 9.79254237976727)	0.241628483292278 (0.105635755706924 to 0.388833421191953)
Contact dermatitis	Female	81678966.2394421 (64114417.9003204	753207.322519242 (477124.378017048		3333.5759813133 (2626.42242652199	30.5172220889984 (19.2734901581754		136987828.908341 (107791384.000582	830726.052093036 (662791.225454209		3326.85530667856 (2618.91055095481	30.2843114747425 (19.1717577365724	

		to 101048976.872292)	to 1105020.35656325)		to 4085.63598193387)	to 44.94761569526)		to 167460205.153724)	to 1020871.30244502)		to 4070.23124153319)	to 44.853010415161)	
	Male	66468383.6577058 (51152527.1619048 to 82485235.5599405)	622170.797865627 (389018.692591072 to 934008.595908275)		2774.15440920129 (2169.18874537313 to 3395.00645714932)	25.6677000351402 (16.0897540033618 to 38.1025881553678)		112416896.375013 (87378720.9086001 to 137612873.883217)	815090.539980161 (510416.050995845 to 1051518.43206501)		2802.78588994709 (2182.8658524491 to 3426.86201070071)	25.8155588715591 (16.0937922360008 to 38.5597470415293)	
Decubitus ulcer	Female	851061.47777739 (760880.105552056 to 950042.259274935)	146145.318279704 (105003.82254081 to 193732.330213005)	7823.1515671198 (4762.960685085 to 11219.7715430752)	45.0730422814953 (40.2263582145392 to 50.1168518446077)	7.54259315845245 (5.3828675875018 to 9.91253267229059)	0.454838260846104 (0.272641536856409 to 0.650847293528812)	1731039.1630162 (1558432.68850847 to 1919876.7068505)	1733682.59508413 (1241898.09105726 to 2282838.6931909)	15020.6285637044 (9636.35805024151 to 19161.040401038)	39.7137832316166 (35.7741042334692 to 43.9849549522909)	6.01073348773778 (4.4365933948892 to 7.14288339871894)	0.342463668597645 (0.21990404734039 to 0.436398642920532)
	Male	690883.774008557 (623021.823334794 to 768269.561852936)	121700.473554393 (81912.3469185381 to 194497.029863173)	4780.01235775893 (2533.87616542244 to 8416.36549486984)	46.7336279792408 (41.7447991564498 to 52.7987992688293)	7.81699098415033 (5.16635889253068 to 12.5116540517196)	0.410822401636632 (0.209794807516148 to 0.726306722843691)	1439756.76983691 (1309186.37583712 to 1584574.14492567)	1772053.53164832 (1261969.41161846 to 2355814.79853407)	9367.94440757352 (5648.09465999232 to 14812.2057268039)	43.3405180412282 (39.2866961581377 to 48.1034958216678)	6.33889687895714 (4.48224058995908 to 8.89239139355338)	0.310616151780857 (0.185308891100128 to 0.494351656021539)
Dermatitis	Female	141298460.398978 (123299207.143249 to 161685513.519011)	4255676.51498011 (2473627.44092832 to 6741611.71194837)		5556.62511052459 (4814.52609664378 to 6358.0443405649)	157.998910677355 (92.6894391580556 to 248.917904644829)		223064365.690971 (192251672.592991 to 255879663.654629)	445666.718550955 (213453.332337001 to 794536.594776439)		5563.24978219179 (4818.03814594433 to 6368.63914303838)	151.790960845631 (89.0930830387159 to 239.062704575596)	
	Male	123588603.810902 (108075794.624656 to 140683997.806212)	3142007.87815814 (1834939.14756987 to 4965960.45395269)		4855.01069347212 (4210.22114459539 to 5512.01378518967)	114.176733233294 (67.2877650151005 to 178.898717360183)		195380172.54414 (169030945.794957 to 222543807.034809)	340083.749671759 (162628.632171465 to 610492.929431344)		4924.00972395899 (4270.56095081146 to 5599.55902347194)	111.840990334187 (66.2202086679382 to 174.98260212612)	
Fu	F	4904	9431		1973	37.23		8119	1894		2046	39.72	

ngal skin diseases	emale	75184.36205 (442826205.998302 to 541696607.970849)	27.8018452 (384845.081718927 to 1992389.11095058)		4.0082610134 (17925.3639592472 to 21738.7215143451)	69163670356 (15.2209371017666 to 78.9959067447421)		96579.358417 (736821920.723577 to 894163063.1207)	543.11256147 (938789.962337551 to 3411656.58258343)		8.2564608993 (18579.2410313645 to 22505.9298934699)	26014887346 (16.3049871650111 to 83.8894695177965)	
	Male	511641273.37294 (459169907.276696 to 570900099.559441)	1025020.24609305 (414735.272703859 to 2155478.78823581)		21219.7341244924 (19264.785081942 to 23509.9677672394)	41.1013684358178 (16.7141660632468 to 86.7902863945819)		834600377.069998 (755679903.053001 to 921636904.446979)	1710087.93923548 (834752.248984259 to 3087260.48349226)		22075.8229770987 (19998.1466270687 to 24357.2631074197)	44.2605444831236 (18.0757129343656 to 92.4381019158097)	
Other skin and subcutaneous diseases	Female	208327358.032642 (203628908.86388 to 213408221.852009)	1135711.95449341 (564965.715465747 to 2046508.15873973)	1283.16495430116 (888.807941128922 to 2494.75321405247)	8521.65252300293 (8336.07683943685 to 8724.86624462424)	46.3950272790838 (23.1798861737819 to 83.6601384851376)	0.0647660552742699 (0.0435393288361134 to 0.131871906940672)	350452669.460853 (342643757.79475 to 359090470.090769)	1553045.18926941 (638723.36555485 to 3289174.5514216)	2207.44297618301 (1581.46699063066 to 3479.94257690751)	8616.47061911289 (8423.06564203747 to 8828.85338564151)	46.6021925880681 (23.0763602095675 to 83.9979559730331)	0.0513106775793006 (0.0367546189298532 to 0.0803506376468055)
	Male	186699530.754927 (182325270.489861 to 191436389.147587)	1020162.38682542 (504610.777977794 to 1839941.91507411)	905.625733827274 (583.304714129196 to 1660.44982845686)	7918.45016566595 (7746.70820941115 to 8107.1854794811)	43.17355985073 (21.515726876268 to 77.4618866157506)	0.0622788105925054 (0.0369558373031428 to 0.121064370985622)	316327703.295253 (309203712.024574 to 324107358.054065)	1671384.76165931 (683340.67375611 to 3495500.40816008)	1399.18409417222 (919.380304354715 to 2165.33634124282)	8124.15508887176 (7943.30866158977 to 8319.62408497579)	43.9154692152085 (21.450476406854 to 79.1954340082449)	0.0438730013223591 (0.0279650783893049 to 0.0694440314962694)
Pruritus	Female	18895655.6079187 (16901557.3260696 to 21067853.5459408)	259673.031766776 (124872.945272956 to 461783.246483682)		753.839232111069 (673.33622069456 to 840.419107489577)	10.3566604739779 (4.98440627837572 to 18.4641278133883)		32204119.9564256 (28687476.675404 to 35971020.9802684)	5795237.10862303 (3416569.72552102 to 9095475.94819538)		801.098824393697 (715.751350048189 to 893.321845713961)	11.081212449144 (5.29922233954525 to 19.7840877275364)	
	Male	14458063.6323522 (12915437.3329713	197335.716702745 (95295.4319929949		586.477402697536 (524.764396776328	7.98842102016208 (3.85429501873274		24803900.4127789 (22118433.9738226	4268554.33748491 (2537370.38660166		633.093845029382 (566.25731235012	8.66267032482564 (4.15236128554367	

		to 16170158.2466868)	to 354356.233218576)		to 652.898075269096)	to 14.343668381189)		to 27757269.7695771)	to 6677479.01183409)		to 703.313265751399)	to 15.5680484911327)	
**Psoriasis**	Female	1837365.30049653 (1774853.07250508 to 1900618.94356303)	1374225.5259121 (973757.111924087 to 1821723.0977614)		72.5928989581178 (70.0346152608487 to 75.0531468787069)	56.1030758670928 (39.9144731923451 to 74.483910214868)		2306753.18640849 (2224308.00020535 to 2384894.19370706)	262014.936249816 (193433.195359768 to 311432.1957846)		57.7630619433127 (55.7855010555601 to 59.6717465401422)	42.5129666210377 (30.4011109955269 to 56.0906860808339)	
	Male	1815871.01401481 (1751121.00032867 to 1878135.09532531)	1358429.99899301 (967843.939956757 to 1808969.53355819)		71.8269539263997 (69.2702967439582 to 74.3000225069235)	57.2826188502058 (41.0245031377714 to 76.0000281963819)		2315840.51235877 (2232985.91665356 to 2395283.20220027)	219407.825507962 (153440.097167596 to 303779.683891406)		57.8272498169127 (55.7927868605371 to 59.7983466566626)	44.2103186853295 (31.4960271071392 to 58.7278986670052)	
**Pyoderma**	Female	313515903.616208 (305053857.642362 to 324047546.326314)	616707.837399905 (384896.234419571 to 874230.907793067)	13073.702627156 (8849.28325863401 to 17192.2373039744)	11737.4710138323 (11432.2805573272 to 12106.0867625335)	23.2291609106811 (15.1554009880691 to 31.9798944062759)	0.591542366054219 (0.41944912701418 to 0.755736474110913)	489430021.733095 (477274304.781232 to 504735700.426465)	1241587.0870858 (778721.764308059 to 1835000.43303607)	28169.8462239292 (21640.1535306075 to 36434.130981459)	12846.1014024488 (12513.1045421003 to 13255.9602008605)	21.413825619426 (16.9351762903433 to 26.3614506298645)	0.666407430399784 (0.512883734733827 to 0.853974578222221)
	Male	385162305.983406 (374496393.27741 to 398025693.06511)	607763.083948388 (315843.839168583 to 848194.567006923)	13110.8159802602 (6480.86178811455 to 16930.2059286454)	14467.081237115 (14083.1784322346 to 14941.3602952641)	24.9199737638992 (13.2595066190928 to 33.2579693897331)	0.744115681039082 (0.38010218618055 to 0.926207876089278)	601798065.271065 (585847829.679839 to 621119890.82243)	1034349.44701698 (643347.943627931 to 1550040.43594287)	24288.0849501357 (14046.9162924267 to 32519.2700661997)	15459.4860749739 (15052.8147322806 to 15951.2874177512)	21.6039888442404 (13.4875717417416 to 27.9199663728693)	0.711465729671276 (0.412428680868674 to 0.971636482796523)
**Scabies**	Female	211772773.400402 (184474126.314446 to 242170947.62168)	1806839.98030617 (993264.509608959 to 2886989.28903851)		7613.57143889973 (6687.17633643955 to 8630.89407777922)	64.8766454021386 (35.7265545013986 to 103.336067220102)		279252935.181576 (246602144.328858 to 312974470.337309)	274178.103809761 (170470.976981921 to 329311.810989739)		7400.91168448827 (6539.35101273154 to 8333.32884081102)	63.1607735481371 (35.0250887541055 to 100.403061893586)	
	Male	2203	1890		7744.	66.34		2858	2772		7443.	63.86	

	ale	05944.516066 (191540255.002561 to 251974747.53851)	277.76103685 (1032763.37178525 to 3022162.90403189)		86241211945 (6796.5057388313 to 8778.45305788335)	98121945149 (36.3633247565677 to 105.955858141666)		01332.167278 (251822074.504944 to 320844229.890521)	67.907604219 (154806.467905439 to 370496.206413624)		50226341468 (6554.61833469879 to 8381.40215256876)	78079878598 (35.3160786119612 to 101.454185131966)	
**Seborrhoeic dermatitis**	Female	48176737.0179176 (44286414.7592623 to 51937415.6880959)	96348.4407037705 (54930.8677793922 to 153080.677298663)		1809.23711325909 (1665.24175230576 to 1946.79701636237)	3.75496574911013 (2.13500269993674 to 5.94914564951685)		71563417.67094 (65881593.6703262 to 77033239.1862326)	1104904.15590279 (870490.831393742 to 1324182.07267899)		1845.85249788653 (1697.65650403149 to 1985.53259552071)	3.85474123757315 (2.19292472372746 to 6.13676986667169)	
	Male	49375816.5959458 (45347923.9965452 to 53417092.2679782)	98815.8143509144 (55987.7386592177 to 157603.205521878)		1808.51626128259 (1671.46401900367 to 1949.37788424681)	3.91992208174396 (2.22863898215568 to 6.20929941572947)		73030376.9677401 (67347552.3420942 to 78892566.1290182)	1092358.44758437 (719913.141045308 to 1385397.06219394)		1854.53441015265 (1714.90514769573 to 2002.6223586668)	4.01455193873113 (2.279790614637 to 6.42242334873466)	
**Skin and subcutaneous diseases**	Female	1549928705.30813 (1489664044.82574 to 1613836872.63217)	16457067.8844771 (11094506.4036272 to 23712643.4147156)	26448.401505672 (20521.309096693 to 35415.8716027831)	59904.1137853207 (57651.6737921188 to 62306.1585383993)	614.935533154554 (416.525255527624 to 889.189638852154)	1.31044851378025 (0.992540904908845 to 1.75766603203398)	2412824491.39552 (2323159191.19635 to 2509074646.14454)	4401491.10565404 (2345112.01393679 to 7388349.69741908)	55151.6479884197 (40851.0030534703 to 69564.6972657737)	61816.9502492455 (59497.6926295863 to 64330.1558731717)	597.616173430184 (398.896549642935 to 879.975778778879)	1.28677312078556 (0.959307955435512 to 1.61573357370516)
	Male	1577566224.69372 (1512141100.42803 to 1645386050.23431)	14279089.4773903 (9564879.67348891 to 21006830.4286493)	22833.1452308429 (15370.6819191381 to 31378.6416435543)	61562.1550769486 (59207.4668824658 to 64168.4083528376)	533.177878795276 (361.804325855184 to 780.329734434816)	1.44218983859475 (0.996189799414997 to 1.99746673248181)	2446443162.05073 (2349576424.39157 to 2548351122.13091)	3078594.01715317 (1641708.71784347 to 5176294.567883)	43370.0190142754 (27084.1659437086 to 59564.5787696149)	63624.1813799758 (61120.2672496384 to 66336.0797341713)	522.240292145769 (348.033924192561 to 774.47840177038)	1.30758336606677 (0.816770068352016 to 1.81928529048448)
**Urticaria**	Female	50319169.3438286 (44158202.4818016	1702090.34480752 (1112433.88413041		1827.15515327825 (1611.1242142962	61.7152453828179 (40.4707083676959		67526590.557087 (59700060.5356395	391945.181316105 (247006.17688876		1815.88261963671 (1601.47991826947	61.5081886269085 (40.443450094066	

		to 57244446.5300611)	to 2468057.84769355)	to 2065.91095320286)	to 88.2730370529397)	to 75960951.7152063)	to 579526.885068159)	to 2055.50425961523)	to 88.783542855704)
	Male	34803978.5187417 (30295929.0109664 to 39857355.4321227)	1187604.94713872 (776134.573473105 to 1720442.28476193)	1222.47348250416 (1076.32293219234 to 1387.70461661332)	41.6394195498985 (27.3411093489136 to 60.0708283609114)	47182321.6260962 (41660262.986764 to 53422817.0647009)	208625.189610337 (130722.600278083 to 311256.847742076)	1245.28783884857 (1096.79201617235 to 1413.79131389624)	42.5241316667324 (27.7858285581562 to 61.6704280228482)
**Viral skin diseases**	Female	39906965.2228664 (38074723.0995586 to 41749067.217643)	1859830.58784612 (1188619.27704623 to 2772312.78204486)	1393.38240552958 (1331.45240259155 to 1454.81760992075)	66.277617107277 (42.4985217023173 to 98.9099175275626)	48476274.4586546 (46429016.161189 to 50555301.563716)	2746363.50203152 (1654153.58308231 to 4343545.0455919)	1371.87838804017 (1310.29649425099 to 1435.22331466664)	62.8394592468474 (40.3129543973351 to 94.0818432545854)
	Male	40866652.4027856 (39137576.3026877 to 42673868.268977)	1945878.40473119 (1234883.00123384 to 2914338.65351999)	1376.64462457541 (1321.71158138526 to 1433.29001272769)	67.4724536352543 (42.9180637783793 to 100.698262373035)	50473288.2016022 (48531752.8256388 to 52451354.2374546)	2208834.16863646 (1320192.49848297 to 3510691.00801707)	1364.61587203207 (1310.04491954507 to 1420.67710109486)	64.9402800920133 (41.3459954565117 to 96.7817127056757)

Abbreviations: UI, uncertainty interval.
